# Intra-settlement scaling across the urban–rural continuum: material stocks, service provisioning, and greenhouse gas emissions in the contiguous United States

**DOI:** 10.1007/s44498-026-00125-w

**Published:** 2026-07-03

**Authors:** Yiwei Yang, Benedikt Grammer, Rafael Prieto-Curiel, David Frantz, Helmut Haberl, Dominik Wiedenhofer

**Affiliations:** 1 Department of Economics and Social Sciences, Institute of Social Ecology, BOKU University, Schottenfeldgasse, 29, 1070 Vienna, Austria; 2 Department of Coevolution of Land Use and Urbanisation, Max Planck Institute of Geoanthropology, 07745 Jena, Germany; 3 Complexity Science Hub, 1030 Vienna, Austria; 4 Geoinformatics – Spatial Data Science, Trier University, 54296 Trier, Germany

**Keywords:** Intra-settlement scaling, Grid-based analysis, Building and mobility infrastructure, Material stock, Greenhouse gas emissions, Population density

## Abstract

Research on urban scaling laws suggests that cities with larger populations tend to be more resource-efficient, yet whether these findings hold across spatial scales, varying definitions of ‘urban’ and the entire rural–urban continuum remains unresolved. Herein, we leverage high-resolution maps for the entire contiguous United States of America to examine how built environment material stocks, service provisioning, and operational greenhouse gas (GHG) emissions scale with population size across the country. Results show that residential building footprints, i.e., the ground area covered by buildings, useful floor area, and material stocks scale moderately sub-linearly (scaling exponent *β* in the [0.82, 0.89] interval). Non-residential buildings and mobility infrastructure (area, materials) scale more strongly sub-linearly (*β* in the [0.42, 0.74] interval). These patterns are robust across sensitivity tests. Furthermore, people living in high-density urban areas require less than half of per capita building footprints, mobility infrastructure area, total infrastructure mass, and residential on-road GHG emissions, compared to residents in low-density rural areas, demonstrating substantial environmental benefits of dense settlements.

**Supplementary Information:**

The online version contains supplementary material available at 10.1007/s44498-026-00125-w. Supplementary Information SI1. This Supplementary Information contains supplementary methodological details, additional explanations, robustness tests, figures, and results, including Supplementary Notes S1, S20, Supplementary Figures S1, S18, Supplementary Tables S1, S24, and Supplementary References. Supplementary Information SI2. This Supplementary Information file provides the numerical data underlying Tables S3, S4, S10, S11, S19, and S23 reported in Supplementary Information SI1.

## Introduction

Buildings and mobility infrastructure constitute one of the largest, most enduring material stocks in the technosphere, driving a major share of global resource extraction and material throughput while locking societies into long-term patterns of energy use and greenhouse gas (GHG) emissions (Galbraith et al., 2025; Haberl et al., 2025). A critical, yet often overlooked consideration is how spatial patterns of settlements and populations shape resource intensity and service provisioning of the built environment (Creutzig et al. 2016; Haberl et al., 2023; Prieto-Curiel et al., 2023). Buildings and mobility systems co-evolve with population distribution and socio-economic interactions. Where and how people live and work could therefore have large effects on resource requirements, land use, and GHG emissions (Duro et al., 2024; Seto et al., 2016).

To address these issues, research on urban scaling laws has begun to uncover systemic relationships between urban population size and various socio-ecological aspects of cities (Zhao & Wu, 2025; Zhou et al., 2022). Specifically, scaling laws delineate the relationship between population size and various urban attributes as *Y*=*Y*_0_*P*^*β*^. Here, an urban attribute *Y* scales with city size, quantified by its population *P*, and the exponent *β* determines how *Y* responds to changes in* P* (Bettencourt et al., 2007). Three characteristic patterns are observed: super-linear relationships (*β* > 1) for many socioeconomic outputs, especially innovation and income (Arbesman et al., 2009); sub-linear scaling (*β* < 1) for physical infrastructure inputs (Batty, 2008); and near-linear scaling (*β* = 1) for individually allocated quantities. These patterns suggest that larger cities generate more innovation per capita, deliver services with less infrastructure per person, and maintain nearly constant per capita individual needs. Recent studies extend this framework to patterns of resource use, waste (Lu et al., 2024; Qu et al., 2023), and CO₂ emissions (Hong et al., 2022; Rao et al., 2023), positioning urban scaling as a useful lens for assessing and guiding sustainable urban development.

However, existing city-level approaches on these systemic scaling relationships lack the spatial and thematic granularity needed to understand scaling patterns within and beyond cities. Urban scaling captures aggregate, average city-level relationships and therefore cannot represent the marked heterogeneity within urban areas (Arcaute & Hatna, 2020; Rybski et al., 2019). Gains from concentration and the economies of scale associated with urban scaling are unevenly distributed across residents and are often skewed towards specific groups (Arvidsson et al., 2023; Heinrich Mora et al., 2021). As such, city-wide averages conceal large disparities at individual and neighborhood scales. Moreover, the widely applied “one-city-one-value” approach assumes that the entire urban area is the primary and only meaningful unit of analysis (Barthelemy, 2019). While urban areas receive substantial attention, it is obvious that cities are embedded in larger landscapes and metabolic hinterlands with which they are connected through infrastructure systems. Cities are not only linked to sprawling suburban and rural settlements, but also to sparsely populated rural regions producing key products (e.g., food) mostly consumed in cities. Carefully delineated city boundaries, whether based on population density, commuting zones, or administrative boundaries, artificially partition continuous settlement systems. Scaling exponents are therefore highly sensitive to how urban areas are defined (Arcaute et al., 2015). For CO_2_ emissions, alternative delineations of urban boundaries can even shift scaling exponents from sub-linear to super-linear regimes (Fragkias et al., 2013; Glaeser & Kahn, 2010; Hong et al., 2022; Louf & Barthelemy, 2014; Mohajeri et al., 2015; Oliveira et al., 2014; Rao et al., 2023; Rybski et al., 2016; see Note S1 of Supplementary Information SI1). This boundary sensitivity introduces substantial uncertainty when scaling relationships are meant to inform policy.

Herein, we aim to address these limitations through a novel, spatially-explicit approach looking at scaling relationships within cities, as well as across suburbs and rural areas, following previous suggestions (Carbone et al., 2022; Dong et al., 2020). Fractal city theory underscores a long-standing empirical insight: cities are self-similar. Urban structures such as street networks and building distributions exhibit fractal self-similarity and scaling, with patterns repeating across spatial scales (Batty, 2008; Michael Batty & Paul Longley, 1994), reflecting cities’ capacity to optimize space and resources through self-organization (Chen et al., 2017). Such patterns are observed not only within city boundaries, but extend to suburban and rural areas (Nguyen et al., 2022). Recent work further suggests that cities worldwide share a fundamental internal structure: intra-city distributions of population, road networks, and carbon emissions across scales can be interpreted as joint fluctuations of spatially dependent random variables (Hendrick et al., 2025). Viewed in this way, the urban landscape grows from the bottom up, which leads to a central hypothesis: fine-grained properties, at least those infrastructure-related attributes tied to urban form, may scale with population across the continuous fabric of human settlements. Such fine-grained relationships are not independent but naturally related to classical city-scale scaling laws. Exploring this hypothesis increases the spatial resolution at which policy and planning strategies can be designed and evaluated.

To test this hypothesis and conduct a systematic assessment of material stocks, service provisioning, and related GHG emissions from buildings and mobility infrastructure, we leverage recent advances in remote sensing and big-data analysis. We assemble datasets for the contiguous United States (contig. U.S.), including 10 m-resolution remote-sensing maps of all buildings and mobility infrastructures, together with 1 km-resolution gridded building and on-road GHG emissions from Vulcan and DARTE. Building on these datasets, we implement a grid-based scaling model that treats grid cells (primarily 1 km-by-1 km) covering the entire contig. U.S. as observations. We then quantify scaling relationships between grid-level population and 18 indicators representing service provision from buildings and mobility infrastructure, their associated material stocks, as well as housing- and transport-related GHG emissions across the full urban–rural gradient. Through stepwise empirical tests, we examine whether fine-grained scaling relationships emerge and evaluate their robustness to outlier removal, urban–rural classification, regional variation, and spatial aggregation. Finally, we compare the grid-based approach with the conventional city-based approach and discuss the potential explanation underlying sublinear exponents across spatial scales. Our findings reframe the view that scaling laws arise only at the city or functional urban area level. They also link citywide sustainability targets to neighborhood-level levers and provide evidence to guide locally grounded, incremental pathways towards sustainable urban development.

## Materials and methods

### Datasets

This study examines the Contiguous United States (contig. U.S.), using a uniform grid system across all data sources to define the analytical units, with most analyses conducted at a 1 km resolution. All datasets are summarized below, with detailed descriptions of the data sources and processing provided in Note S2 of Supplementary Information SI1.

#### Population

We used the 2020 census-block level population data from the U.S. Census Bureau (The United States Census Bureau, 2024). Census blocks represent the smallest geographic units for complete population enumeration (Nancy A. Potok et al., 2020), covering all urban and rural residential areas nationwide and independent of other datasets used in this study. We converted census-block-level vector data into 1 km-by-1 km grid using area-weighted apportionment and aggregated values within each cell to obtain per-cell population totals.

#### Built environment material stock

We used the open dataset described in Frantz et al. (2023), which provides 10 m resolution estimates of area, volume, and material mass for detailed building and mobility-infrastructure categories across the contig. U.S. Following the source taxonomy, we grouped buildings into residential (single-family, multi-family, lightweight structures) and non-residential (commercial, industrial, high-rise, skyscraper) categories. We aggregated these data to a uniform 1 km-by-1 km grid to derive per-cell material stock estimates used in this study.

#### Built environment spatial dimensions

These include building footprint (land area occupied), building gross floor area (sum of all floor areas), street area, and total mobility infrastructure area (combined above- and underground mobility facilities). Building footprint, street area, and total mobility infrastructure area were taken directly from the source dataset (Frantz et al., 2023). Building floor area was derived from the same source through calculations across data subsets. Specifically, building height was estimated by dividing building volume by footprint, and floor counts were calculated by dividing building height by assumed average floor heights, namely 3.0 m for residential buildings and 3.6 m for non-residential buildings. The justification for these assumptions and their potential influence on the scaling results are discussed in Note S3 of Supplementary Information SI1. Total floor area was computed as footprint multiplied by the number of floors. All calculations were performed at 10 m resolution and subsequently aggregated to a 1 km resolution.

Importantly, several indicators derived from the above datasets are closely connected but not interchangeable. For example, building mass and building floor area, as well as infrastructure/street area and mass, are generated through different methodological pathways rather than being directly derived from one another as simple multipliers. Although these indicators are theoretically related, they capture distinct dimensions of infrastructure demand, including land occupation, usable built space, and material stock. Details of the derivation procedures are provided in Note S2 of Supplementary Information SI1.

#### Building and on-road GHG emissions

Building-related GHG emissions were taken from Vulcan Version 3.0 (Gurney et al., 2020), which reports fossil-fuel CO₂ at 1 km resolution for the contig. U.S. We used 2015 residential, commercial, and industrial sector data. On-road CO₂ emissions came from DARTE2017 (Gately et al., 2019), which provides annual 1 km estimates from 1980–2017. DARTE employs a bottom-up method, yielding finer on-road estimates than Vulcan. For consistency, we converted Vulcan’s metric tonnes of carbon (tC/year) and DARTE’s kilograms of CO₂ (kg CO₂/year) to metric tonnes of CO₂-equivalent (tCO₂e/year) before analysis.

#### Urban boundaries

We adopt the Global Human Settlement Layer’s Urban Centre Database (GHS-UCDB; Pesaresi et al., 2024), and the U.S. Census Bureau’s Urban Areas Dataset (USCB-UADS; the United States Census Bureau and the U.S. Department of Transportation, 2024). We additionally incorporate the Morphological Urban Area (MUA; Taubenböck et al., 2019), which delineates more contiguous urban extents by excluding peripheral areas, and the GHSL–OECD Functional Urban Areas (GHS-FUA; Schiavina et al., 2019), which defines broader functional regions based on commuting flows. These datasets contain 322 (GHS-UCDB), 2,586 (USCB-UADS), 119 (MUA), and 258 (GHS-FUA) urban units, respectively. For each area, total population and key indicators were aggregated within urban boundaries. Specifically, to ensure spatial consistency, the coordinate reference system of the 1 km-by-1 km gridded data is used as the reference, and all urban boundaries are reprojected accordingly. Urban boundaries were rasterized on an aligned grid, where cells were assigned to the urban class if their centroid was within the polygon boundaries.

### Grid-based scaling model

#### Model formulation

The conventional urban scaling law relates city size, usually measured by population *P*, to urban attributes *Y* through the scaling exponent *β*. It is expressed as:

1 $$\begin{aligned} &Y = Y_{0} P^{\beta } ,{\text{or in logarithmic form}},\,\\& \log Y = \log Y_{0} + \beta \log P.\end{aligned}$$

Here, we employ a grid-based scaling approach using gridded urban–rural cells instead of cities as observational units (Eq. 2). Lower case notation denotes grid-cell level relationships, in contrast to the uppercase notation used for city level scaling, as

2 $$\begin{aligned} & y = y_{0} \rho^{\beta } ,{\text{or in its logarithmic form}}\,\\ &\log y = \log y_{0} + \beta \log \rho\end{aligned}$$

Here, ρ denotes the total population or population density at the grid-cell level, which are mathematically interchangeable in a grid-based framework (see explanation below and Note S4 of Supplementary Information SI1). ${y}_{0}$ is a normalization constant, β is the scaling exponent, and y represents an infrastructure-related variable at the grid-cell level. In this study, y comprises 18 indicators grouped into three categories: (1) service provisioning, including building footprints and floor areas (total, residential, and non-residential), as well as street and total mobility infrastructure area; (2) material stocks, including total, residential, and non-residential building material mass and mobility infrastructure material mass; and (3) GHG emissions, including residential, commercial, industrial building emissions and on-road emissions.

Units of these indicators are not emphasized, as logarithmic transformation normalizes scale differences across measurement units; unit conversion therefore shifts only the intercept. We estimate *β* and *R*^2^ from the log-linear specification using ordinary least squares (OLS) on log-transformed variables, with *R*^2^ computed in the standard manner.

Importantly, the grid-based approach inherently accounts for density. Although we use total quantities (ρ and *y*) within each grid cell, the fixed and equal cell area ensures that these totals implicitly represent density measures. We further confirm that using implied density (total value per cell) or explicit density (total value divided by grid area) yields identical scaling exponents (see Note S4 of Supplementary Information SI1). In practical terms, *y*_0_ and *β* jointly characterize how differences in population density ρ relate to the density of the response variable *y*.

#### Uncertainty analyses

To evaluate the robustness of the scaling relationships, we conducted uncertainty analyses addressing both structural and input-related variability, including grid placement effects, residential estimation uncertainty, and potential biases from unmodeled spatial dependence. Due to space constraints, detailed descriptions are provided in Notes S5–S7 of Supplementary Information SI1.

### Classified linear regression by urban boundaries

To assess whether urban and non-urban contexts follow the same scaling relationship, we conduct separate grid-based scaling analyses for urban and non-urban grid cells across the contig. U.S, applying the log-linear model in Eq. 2. Urban boundary vectors are rasterized to 1 km-by-1 km grids. Each cell is assigned to the class according to the majority area rule. The resulting grids are aligned with population rasters to distinguish cells inside and outside urban boundaries.

### Windowed linear regression

To assess how scaling exponents vary across regions with distinct geographical characteristics, we subdivide the contig. U.S. into 15,600 subregions of 30 km-by-30 km (“windows”) and conduct grid-based scaling analyses within each “window”, excluding grid cells with population density ≤1 person/km^2^. The regression results are mapped back to their corresponding windows. Each window contains a maximum of 900 data points. However, a minimum of 10 observations is required for estimation. Windows not meeting this threshold are excluded. The rationale for selecting the 30 km-by-30 km window size and the minimum-observation threshold is provided in Note S8 of Supplementary Information SI1.

### Variation of the grid-based scaling law across different grid cell sizes

We conducted regression analyses at grid resolutions ranging from 1 to 20 km to examine the impact of spatial aggregation on scaling exponents. This was done by aggregating all 1 km-by-1 km gridded maps to coarser resolutions and estimating the log-linear model in Eq. 2 at each scale.

### Quantifying per capita variations under the grid-based scaling law

To analyze per capita variations, Eq. 2 can be reformulated as:

3 $$\frac{y}{\rho }={y}_{0}{\rho }^{\beta -1}$$

In sublinear scaling where *β*—1 < 0, higher population density is associated with lower per capita values (*y/*ρ). Suppose the population density in grid A is *x* times that of grid B, i.e., ρ_*A*_ = *x* ρ_*B*_. Then, we derive:

4 $$\begin{aligned} \frac{{\left( {y_{A} /\rho_{A} } \right)}}{{\left( {y_{B} /\rho_{B} } \right)}} = \left( {\frac{{\rho_{A} }}{{\rho_{B} }}} \right)^{\beta - 1} = x^{\beta - 1} , \\ {\text{or equivalently}}\frac{{y_{A} }}{{\rho_{A} }} = x^{\beta - 1} \cdot \frac{{y_{B} }}{{\rho_{B} }}\end{aligned}$$

Normalizing ${y}_{B}/{\rho}_{B}$ to 100%, the per capita value in grid A becomes ${x}^{\beta -1}\times 100\%$. Because *β*—1 < 0, ${x}^{\beta -1}$ is a decreasing function of *x*, indicating an inverse relationship between per capita values and population density.

## Results

### Intra-settlement scaling relationships across the urban–rural continuum

**Fig. 1:**
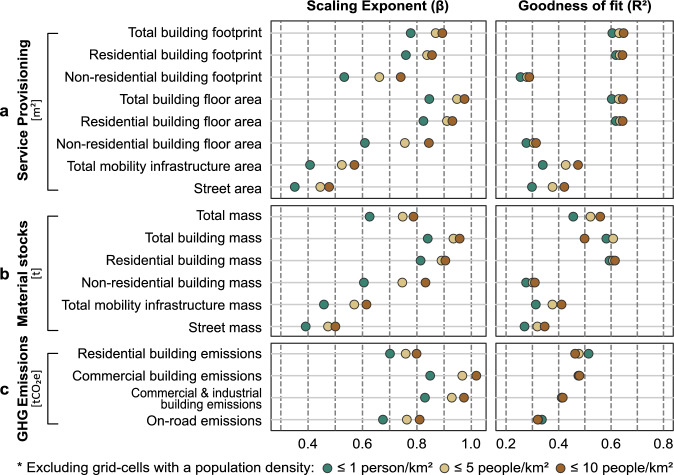
Grid-based scaling relationships between (**a**) service provisioning, (**b**) material stocks, (**c**) GHG emissions, and population across the contig. U.S. Scaling exponents (*β*) and goodness of fit values (*R*^2^) for 18 key indicators, analyzed using 1 km-by-1 km observation units. Colors represent filters for population density. A scaling exponent (*β*) of 1.0 indicates that grid cells with larger populations are associated with proportionally larger values of the variable of interest; a *β* value of 0.5 reflects sublinear scaling, whereby a grid cell with twice the population is associated with only about 1.4 times (i.e., 2^0.5^) the respective variable. Colored points denote various population density filters in terms of people/km^2^. These filtering criteria have been shown to affect both *β* and *R*^2^ values. For detailed regression results, refer to Notes S10 of Supplementary Information SI1. Due to the large effective sample size (n > 5 million), the 95% confidence intervals for *β* estimates are extremely narrow (± < 0.003; see Notes S11 of Supplementary Information SI1 for calculation and results). Error bars are therefore not shown

Our analysis of 1 km-by-1 km grid cells across the contig. U.S. reveals robust intra-settlement scaling laws between population and infrastructure-related variables. Across 18 indicators of buildings and mobility infrastructure, average scaling exponents (*β*), computed by averaging estimates across alternative population density filtering criteria for each indicator, range from 0.42 to 0.94 (Fig. 1). The population-density filter thresholds were chosen to reduce the potential influence of uncertain grid cells with extremely low estimated populations, which may arise from census-data uncertainty or the vector-to-grid transformation process (see Note S9 of Supplementary Information SI1 for details). Our results show that areas with higher population density are associated with a substantially lower per-capita demand for mobility infrastructure and a slightly lower demand for buildings.

We categorize the 18 infrastructure-related indicators into three environmental dimensions: service provisioning, material stocks, and GHG emissions. For service provisioning (Fig. 1a), total building floor area scales nearly linearly with grid-based population, with an average *β* value of 0.92 across different population filters. However, total building footprint (the ground-level horizontal area occupied by the building) shows a more pronounced sublinear relationship, with an average *β* value of 0.84. These findings indicate that in the contig. U.S., denser settlements do not correspond to proportionally larger building footprints or substantially lower per capita floor area. Instead, higher-density areas tend to feature larger building heights. Furthermore, per capita residential floor area remains largely unchanged within high-density areas, whereas that per capita floor area of non-residential buildings is notably smaller (average *β* = 0.74 across different population filters), suggesting more efficient use of shared resources in dense settings. Moreover, mobility infrastructure shows an opposite scaling pattern, with lower scaling exponents: an average *β* of 0.50 across population filters for total mobility infrastructure areas and 0.42 for street areas. These lower exponents reflect smaller per capita requirements for mobility infrastructure in dense areas, consistent with the shorter physical distances between individuals in densely populated regions.

Higher-density areas are also characterized by smaller per capita material stocks (Fig. 1b). Compared to grid cells with lower population density, those with twice the population contain about 1.9 times the total building material mass (average* β* = 0.91 across different population filters), 1.5 times the mass of mobility infrastructure (average* β* = 0.55), and 1.7 times the combined mass of both types of structure (average* β* = 0.72). Noteworthily, the similar scaling patterns observed between building mass and building floor area, and between infrastructure/street mass and area, may reflect intrinsic connections among different dimensions of the built environment, rather than indicating that these indicators are directly substitutable (see Note S2 of Supplementary Information SI1).

We identified a sublinear relationship between population density and GHG emissions from buildings and mobility infrastructure during their operational phase (Fig. 1c). Grid cells with twice the population size contain 1.7 times the GHG emissions from residential buildings and on-road transportation (average* β* = 0.75 across different population filters), compared to lower-density cells. Commercial and industrial emissions in these grid cells are 1.9 times those of their lower-density counterparts (average* β* = 0.91). In high-density areas, buildings tend to exhibit higher volume-to-surface area ratios, leading to lower per-unit-area heating and cooling demand. Shorter travel distances and greater availability of public transportation contribute to lower on-road emissions. By contrast, emissions from commercial and industrial sectors exhibit a near-linear relationship with population density, reflecting fixed energy demands and high operational intensity in these sectors. These results highlight sector-specific patterns in the relationship between population density with GHG emissions.

### Intra-settlement scaling in urban versus non-urban areas

Separate grid-based scaling analyses are performed for urban and non-urban grid cells across the contig. U.S. using two independent urban boundary definitions described in the Methods section (GHS-UCDB and USCB-UADS). The resulting scaling relationships are compared with those from the full sample, which include both urban and non-urban grid cells (Fig. 1).

**Fig. 2:**
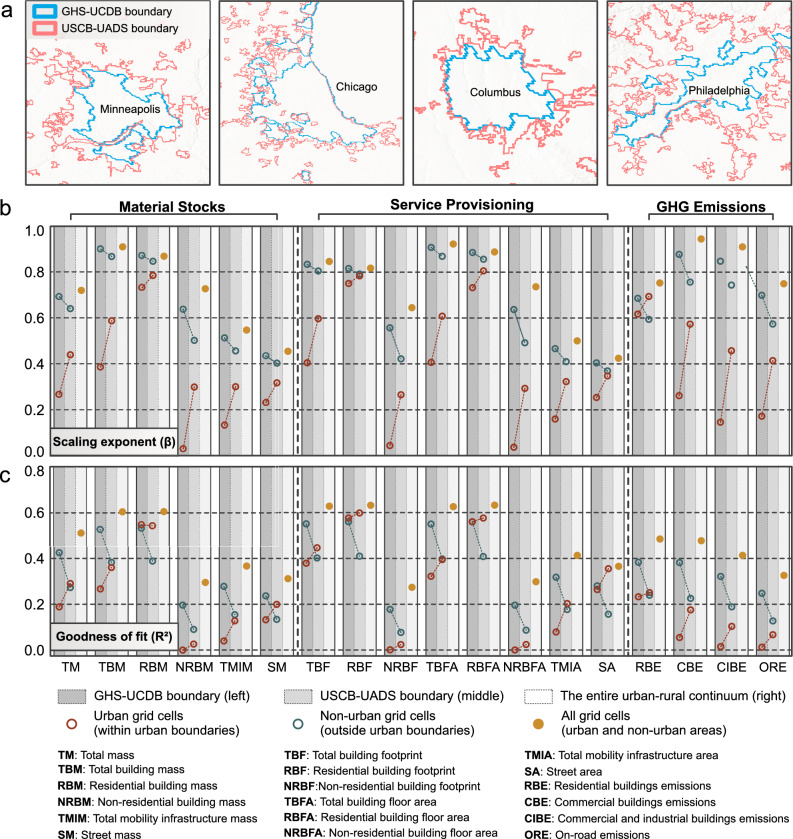
Effect of spatial extent of city boundary definitions on scaling exponents in urban and non-urban contexts. **a** Comparison of urban area extensions, exemplified by four American cities, based on two distinct boundary definitions. The cities shown here are selected solely for visualization and comparison purposes. The GHS-UCDB boundary (blue) defines urban centers based on specific population thresholds and built-up surface areas, with a minimum required population of 1,500 people (urban cells *n* = 93,034; non-urban cells *n* = 5,805,628). The USCB-UADS boundary (pink) encompasses densely developed areas, with a minimum population of 5,000 people (urban cells *n* = 276,611; non-urban cells *n* = 5,622,051). **b** Scaling exponents (*β*) for urban, non-urban, and urban–rural context analyses at the 1 km resolution. **c** Goodness of fit values (*R*^2^) for urban, non-urban, and urban–rural context analyses. The data for Fig. 2 can be found in Table S11 of Supplementary Information SI2

The GHS-UCDB delineates smaller, more concentrated urban centers, while USCB-UADS encompasses broader suburban peripheries (Fig. 2a). In urban context analyses, scaling exponents (*β*) and goodness-of-fit (*R*^*2*^) are higher under the USCB-UADS boundary than under the GHS-UCDB boundary (Fig. 2b, c). Conversely, in non-urban contexts, the GHS-UCDB boundary yields greater *β* and *R*^*2*^ values. In both cases, definitions with greater spatial extent are associated with larger scaling exponents and improved model fit.

The highest *β* and *R*^2^ values are consistently observed in the full sample spanning the urban–rural continuum, compared to analyses based on partitioned urban or non-urban subsets. This is likely attributable to the broader range of population density in the full sample, which spans several orders of magnitude and enables more robust statistical estimation of scaling exponents (Note S12 of Supplementary Information SI1). These results suggest that intra-settlement scaling relationships are most clearly expressed across the full urban–rural continuum.

### Spatial stability of intra-settlement scaling laws across the Contig. U.S.

**Fig. 3:**
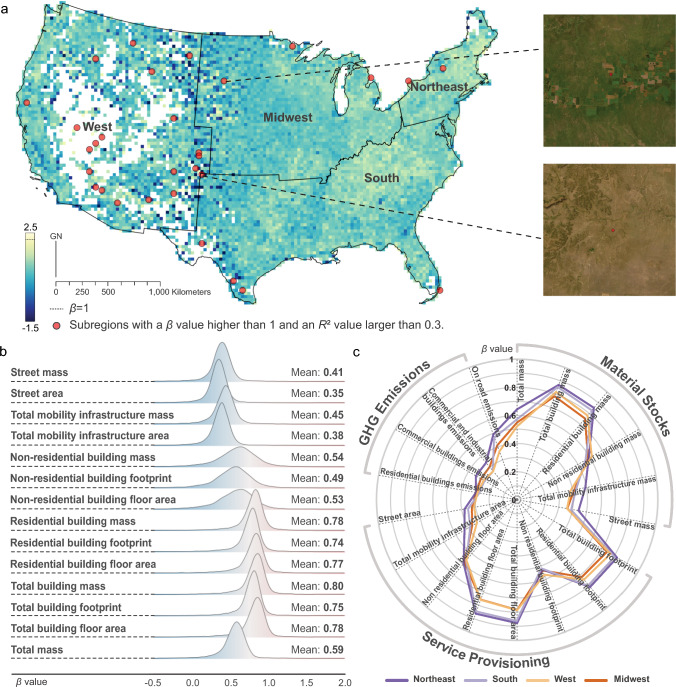
Spatial distribution of grid-based scaling exponents. **a** Grid-based scaling relationship between population and total mass within various subregions. Each red point represents a subregion exhibiting statistically reliable super-linear scaling (*β* > 1, *R*^2^ > 0.3), primarily located in rural or mountainous areas characterized by sparse populations. **b** Statistical distribution of regression results for key indicators related to building and mobility infrastructure. **c** Averaged scaling exponents for 18 key indicators across four major U.S. regions, with each color representing a different major region. The geographical boundaries of each region correspond to the areas shown in panel A. The data underlying Fig. 3 are available on GitHub (https://github.com/socialecologyboku/SociometabolicScaling) and on Zenodo (https://doi.org/10.5281/zenodo.20594106)

Windowed linear regressions are applied to examine regional variation in scaling exponents across 15,600 subregions in the contig. U.S. We present the results of total mass using the population-density filter of ≤1 person/km^2^ as a representative example (Fig. 3a). Results for other indicators and for other population density filters are provided in Fig. S13–S15 of Supplementary Information SI1. Fine-grained scaling exponents vary geographically in a statistically regular manner (Fig. 3b). For each indicator, the empirical distribution of subregional exponents is approximately normal, with mean values closely matching the national estimates.

A few subregions exhibit a super-linear or even negative scaling. These outliers are predominantly located in sparsely populated rural or mountainous areas with disproportionately extensive mobility infrastructure. In these regions, grid-cell population varies only within a narrow range (typically 1–50 people), while infrastructure mass spans several orders of magnitude. This discrepancy results in pronounced numerical disparities and anomalous scaling exponents with weak statistical reliability (*R*^2^ < 0.3).

We compared regional differences across the four major U.S. regions defined by the U.S. Census Bureau. Figure 3c shows mean scaling exponents for 18 key indicators based on windowed regression results across the four major U.S. regions. The Northeast, which is characterized by high economic activity and population density, exhibits higher mean scaling exponents for residential building floor area and mass, residential building footprint, mobility infrastructure area and mass, and non-residential emissions. These patterns suggest relatively greater scaling of individual housing demand and mobility infrastructure with population size in the Northeast. By contrast, for non-residential building footprint, regions with higher per-capita GDP (Northeast and the South), display lower exponents than the Midwest and West, suggesting more intensive sharing of public space.

We further conducted both parametric and non-parametric statistical tests to evaluate regional differences in the estimated scaling exponents across the four major regions (details shown in Note S13 of Supplementary Information SI1). The results show that all indicators exhibit statistically significant regional differences at the p < 0.001 level (Table S13 and S15 of Supplementary Information SI1). Regional variation is more pronounced for residential building-related indicators, especially residential building floor area, than for non-residential building indicators. However, building-related emissions show much weaker regional effects. For mobility-related indicators, regional effects are more evident in total mobility infrastructure area and mass, as well as in on-road emissions.

Overall, although economic and geographical factors contribute to regional variation, the effect sizes indicate that the practical magnitude of these regional differences is generally small to modest and varies across indicators (Table S13 and S15 of Supplementary Information SI1). Therefore, while regional differences are statistically detectable, they do not alter the overall trend of the observed scaling relationships.

### From cells to cities: environmental implications of dense settlements

**Fig. 4:**
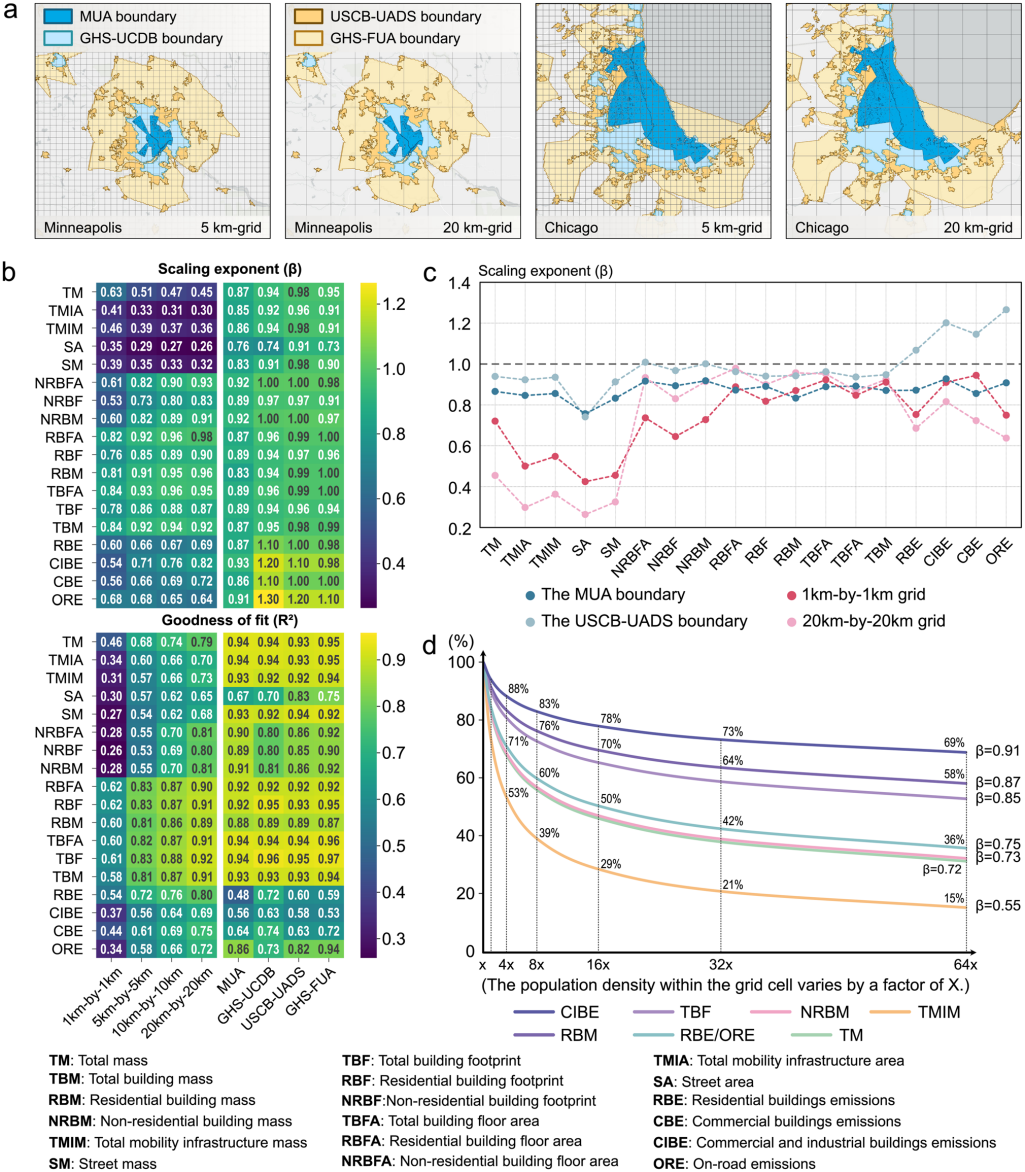
Diverse scaling outcomes under different data aggregation approaches. **a** Examples illustrating different data aggregation methods. Minneapolis and Chicago are used as case examples. They are selected solely for visualization and comparison purposes. The Morphological Urban Area (MUA) delineates highly contiguous and aggregated urban regions while excluding urban–rural transition zones. The GHSL-OECD Functional Urban Areas (GHS-FUA) delineates functional urban regions consisting of a city and its surrounding commuting zone. The GHS-UCDB and USCB-UADS boundaries, previously defined in this study, are also included. The reference extents of the 5 km and 20 km grid systems are shown for comparison. **b** Heatmap showing the scaling exponent (*β*) and goodness of fit (*R*^2^) across different data aggregation methods. **c** Line chart illustrating the contrasting scaling patterns of GHG-emission-related indicators when different urban boundaries are applied, with results for these city-based analyses represented in blue. **d** Scaling curves illustrating per capita values of key indicators across grid cells with different population densities, from a cross-sectional perspective. The horizontal axis represents the population density of spatial units, expressed as a multiple of that of a baseline unit with population density *x*, while the vertical axis shows per capita values of those units as a percentage relative to the per capita value of the baseline unit. Each curve corresponds to a specific indicator. Note: The horizontal axis reflects cross-sectional variation among spatial units with different population densities, rather than temporal changes within individual units

Here, we examine scaling relationships across multiple grid-cell scales and contrast them with conventional city-based scaling laws defined by alternative urban boundaries. This comparison links fine-grained settlement structure to city-level urban scaling patterns and has implications for resource-efficient and sustainable urban development. Figure 4a–b present scaling results under four alternative urban boundary definitions and varying grid-cell sizes, showing that scale dependence is a ubiquitous feature of both grid- and city-based scaling. In grid-based analyses, increasing the grid cell size results in higher *β* and *R*^2^ values for all variables except those related to mobility infrastructure (Note S14 of Supplementary Information SI1). When the grid size reaches 20 km-by-20 km, the scaling exponents for building-related variables converge toward city-based estimates. This convergence is not driven by grid areas approaching typical city sizes: in the contig. U.S., the median urban area is 445 km^2^ for MUA-defined cities and 1,224 km^2^ for GHS-FUA-defined cities, both larger than a 20 km-by-20 km grid cell (Note S15 of Supplementary Information SI1). In city-based analyses, alternative boundary definitions induce fluctuations in scaling exponents; for emission-related variables, different delineations can even produce opposite scaling regimes, consistent with previous findings (Fig. 4c; Note S1 of Supplementary Information SI1).

To interpret the result, we draw on the fractal-based explanation proposed by Molinero and Thurner (2021), which interprets the scaling exponent for a spatially distributed variable as the ratio between the fractal dimension of the measured object and that of the population, where the fractal dimension denotes how fully a geometric object or spatial distribution occupies space across scales. We use this framework as a theoretical interpretation of the observed scaling patterns, rather than treating the fractal dimensions as directly estimated quantities in this study. Further interpretation regarding fractal dimension is provided in Note S16 of Supplementary Information SI1. In this framework, sub-linear scaling arises when the fractal dimension of infrastructure (${d}_{i}$) is smaller than that of the population (${d}_{p}$): ${\beta}_{sub}={d}_{i}/{d}_{p}<1.$ This fractal perspective applies not only at the city scale but also at fine spatial scales, as derived in Note S17 of Supplementary Information SI1.

The observed scale dependence of *β* results from the scale dependence of the fractal dimensions of the measured objects. (1) For building-related variables (footprint, floor area, and mass), the spatial complexity and fractal dimension represented by each cell increase as grid size grows (Carbone et al., 2022). This is not only because larger grids cover broader spatial extents, bringing their fractal dimensions closer to those observed at the city scale, but also because larger grids are more likely to capture remote structures (such as industrial facilities) that may be excluded at finer resolutions by population filters. As the population fractal dimension remains relatively stable across scales, the increase in infrastructure fractal dimensions leads to higher *β* values. This also explains why grid-based exponents are typically smaller than those derived from city-level analyses (see Note S17 of Supplementary Information SI1). (2) As grid size increases, the scaling exponent for street area decreases. Across all grid sizes, the combined exponents for street area and total building footprint consistently sum to approximately 1.13–1.15. This compensatory relationship is further supported by variance-propagation analysis across grid sizes (see Note S18 of Supplementary Information SI1). A plausible explanation lies in geometric constraints: each grid cell has a fixed two-dimensional area within which streets and buildings must share space. This spatial constraint likely induces a trade-off between their fractal dimensions: greater spatial complexity in one structure corresponds to reduced complexity in the other.

Two key implications emerge from these results. First, city-based scaling, particularly when functional urban areas are used as the observational unit, yields scaling exponents for building floor area and building mass are close to 1, indicating equal per capita demand for living and working space regardless of city size. In contrast, grid-based analyses reveal a clear sub-linear scaling relationship, whereby higher-density units deliver services with substantially lower per capita material stocks and GHG emissions (Fig. 4d). This apparent city-scale equality thus results from aggregating two opposing intra-settlement patterns: lower per capita demand in high-density areas and higher per capita demand in low-density areas.

Second, these fine-grained disparities manifest as pronounced per capita differences across settlement units with different densities. Using population-density benchmarks for the contig. U.S.: 50 people/km^2^ for rural (baseline; Note S19 of Supplementary Information SI1), 600 people/km^2^ for suburban (12 × baseline), and 1,800 people/km^2^ for urban (36 × baseline), we find that in terms of service provisioning, people residing in high-density urban units require only 58% of the per capita building footprint and 17% of the mobility infrastructure area of those in rural areas, while suburban residents require 68% and 29%, respectively (see Methods section for calculation details). For material stocks, urban units require only 37% of the per capita building and mobility infrastructure mass compared to rural units, while suburban units require 50%. Regarding GHG emissions, per capita emissions from residential buildings and on-road transport in urban and suburban units are 41% and 54%, respectively, of those in rural units. Per capita emissions related to commercial and industrial buildings in urban and suburban units are 73% and 80%, respectively, of rural levels (see Table S23 of Supplementary Information SI2 for the complete results for all 18 indicators).

Overall, in the contig. U.S., people living in high-density urban areas need only half or less, on a per-capita basis, of the building footprint, mobility infrastructure area, total material mass, and residential or on-road GHG emissions, as well as about one-quarter of the emissions from commercial and industrial activities, compared to people in low-density rural areas.

## Discussion and conclusion

In this work, we proposed a novel grid-based scaling framework that addresses limitations of the conventional “one-city-one-value” approach widely used in the literature. Our novel approach shifts focus from city-aggregate measures based on varying definitions of ‘urban’, to fine-grained analysis across the full urban–suburban–rural continuum, which substantially increases robustness of our findings, as well as their policy-relevance. Using high-resolution geospatial gridded data for the contig. U.S., we reveal that, at the grid cell level, population and 18 infrastructure-related indicators follow power-law forms with pronounced sublinear to nearly linear relationships (*β* in the [0.42, 0.74] interval across different population filters).

Regarding the robustness of our findings, the tests of grid placement effects and uncertainty in residential variable estimations yielded scaling relationships that were nearly identical to the originals (Notes S5–S7 of Supplementary Information SI1). To assess unmodeled spatial dependence, we compared the ordinary least squares (OLS) approach with spatial lag (SLM) and spatial error models (SEM) (Notes S7 of Supplementary Information SI1). These models were implemented at multiple spatial resolutions, including 1 km, 5 km, 10 km, 20 km, and 30 km, for three selected variables: total mass, total building mass, and residential building mass. These variables were selected because they ranked among the highest in the residual spatial autocorrelation analysis. We found that the OLS residuals exhibit mild spatial autocorrelation, with its magnitude varying across indicators and spatial scales. Among the three approaches, SEM more effectively absorbs the residual spatial autocorrelation. Nevertheless, the estimated scaling exponents (β) and model fit (*R*^2^) differ only marginally between SEM and OLS, suggesting that the unmodeled spatial dependence in the main analysis is unlikely to introduce substantial bias into the results or their interpretation (Table S9 of Supplementary Information SI1). Therefore, to maintain comparability between the grid-based scaling model and the conventional city-based model, we retained OLS as the main model specification.

Moreover, we observed relatively lower *R*^2^ values when using 1 km-by-1 km grid cells compared with coarser spatial resolutions (Fig. 4b). However, the near-identical *R*^2^ values across different modelling approaches (Table S9 of Supplementary Information SI1) indicate that these relatively lower *R*^2^ values do not necessarily imply reduced model reliability. Instead, they may reflect an intrinsic property of fine-scale spatial analysis caused by the spatial decoupling between infrastructure and recorded residential population at grid-cell level, and the magnitude of this decoupling varies across indicators and spatial scales. Specifically, the population data used in this study are recorded at residential locations rather than according to daytime activity patterns. In extreme cases, a 1 km-by-1 km grid cell may contain very few recorded residents but a substantial amount of infrastructure, such as in industrial zones and transportation corridors, thereby leading to deviations from the expected scaling regressions. Therefore, lower *R*^2^ values are observed mainly for non-residential and mobility infrastructure-related indicators, whereas residential-related indicators show better fit (Fig. 4b). As grid size increases, spatial aggregation naturally smooths out the effects of spatial decoupling. This aggregation effect typically leads to lower overall variability in the data and, consequently, higher *R*^2^ values. Taking together, scaling exponents and goodness of fit remain robust across alternative grid placements, regression models, and residential variable estimation approaches, indicating that the sublinear scaling of infrastructure is already evident within settlements and is not merely an emergent phenomenon from city-wide aggregation.

We identify three prominent properties of the intra-settlement scaling law: (1) Accounting for the full urban–suburban–rural density gradient is essential for robust estimation; analyses restricted to urban cells alone yield weaker fits. (2) Scaling varies regionally, with empirical exponents approximately normally distributed around means close to national estimates. (3) Scaling is scale-dependent: changing grid size induces modest and systematic shifts in *β*, and as cell size approaches the city scale, exponents for most infrastructure indicators approach city-level values, except for mobility infrastructure. A simple mechanism drives these patterns: sub-linearity arises when the fractal dimension of the population exceeds that of infrastructure. Moreover, planar infrastructure such as street area and building footprints face finite-area constraints and therefore show a trade-off in their scaling.

This study has several limitations that warrant further investigation. (1) The absence of complete time-series data for the variables examined in this study prevents assessment of temporal robustness of the estimated scaling relationships. In addition, the datasets used to derive the emission- and infrastructure-related indicators are not referenced to exactly the same year, although they fall within a close period. This temporal mismatch does not affect the estimation of each individual scaling relationship; however, it may introduce some uncertainty when comparing scaling exponents across indicators. Given the relatively short time interval between the datasets, we expect this influence to be limited. Previous studies also show that cross-sectional urban scaling exponents estimated across systems of cities can remain relatively stable over time (see Note S20 of Supplementary Information SI1; Burghardt et al., 2024; Fragkias et al., 2013; Carleton et al., 2025; Xu et al., 2020; Bettencourt, 2013; Lu et al., 2024; Depersin & Barthelemy, 2018). However, whether intra-settlement scaling relationships exhibit similar temporal persistence remains unresolved and requires further investigation using longitudinal data. Nevertheless, planning interventions might plausibly shift scaling exponents, with sprawling development associated with larger values and compact forms with smaller ones relative to our estimates. As longitudinal emission- and infrastructure-related datasets become more complete, future research should evaluate the temporal stability of grid-based scaling across stages of urban development. (2) Using standardized grid cells as observational units enhances transferability across diverse regions. Although our analyses are grounded in U.S. data, the results provide a benchmark for comparative global research. Expanding broad-coverage, high-resolution socio–resource–environment datasets will be critical for testing grid-based scaling across contexts and spatial scales. (3) At fine spatial scales, “active population” derived from mobile-phone location data may better reflect functional human presence than census-based residential counts (Hanley et al., 2016; Li et al., 2017). Developing national-scale active population datasets would enable their use as primary drivers in our scaling models and potentially improve estimation accuracy.

Overall, this study offers three principal implications for the practice of sustainable settlement development. First, it provides an integrated framework to quantify how population density shapes resource use, service provisioning, and GHG emissions. The comparison across indicators shows that the implications of population concentration are not uniform across different dimensions of the built environment. Building-related indicators generally exhibit sublinear scaling, but their relatively high scaling exponents indicate that grid cells with larger populations still tend to contain substantially greater building footprints, floor area, and material stocks, although the relationship is less than proportional. By contrast, mobility-related infrastructure shows a much stronger sublinear pattern. A 1 km-by-1 km unit with twice the population is associated with only approximately 1.35 (2^0.43^) times the street area, 1.37 (2^0.45^) times the street mass, and 1.46 (2^0.55^) times the total mobility infrastructure mass. This suggests stronger economies of scale in the spatial and material requirements of mobility infrastructure in denser grid cells, consistent with the spatial trade-off between building footprints and street footprints within limited land areas. At the same time, the comparison between material stocks and GHG emissions shows that material efficiency and carbon mitigation should be considered separately. While mobility infrastructure exhibits strong sublinear scaling in both area and mass, on-road emissions show a less pronounced sublinear relationship, with a 1 km-by-1 km unit containing twice the population associated with approximately 1.68 (2^0.75^) times the on-road emissions. This suggests that the material efficiency associated with compact settlement patterns does not necessarily correspond to an equivalent carbon-efficiency advantage in the transport sector; realizing such an advantage also requires broader social, functional, and technical actions.

Second, it reveals pronounced per capita heterogeneity across the rural–suburban–urban density gradient. In the contig. U.S., people living in high-density urban areas (> 1,800 people/km^2^), corresponding to the top 30% of urban cells within GHS-UCDB–defined urban area across the United States, are associated with, per capita, half or less of the building footprint, mobility infrastructure area, total material mass, and residential or on-road GHG emissions compared to those living in low-density rural areas (∼ 50 people/km^2^). Although the total population residing in low-density rural areas is one-third smaller than those living in high-density urban areas (excluding suburban areas), rural areas contain 1.7 times the total material mass, 1.2 times the building footprint, 3.2 times the mobility infrastructure area, and 1.3 times the on-road emissions of urban areas. In absolute terms, this corresponds to tens of billions of metric tons more material mass, tens of billions of square meters larger building footprint and mobility infrastructure area, and hundreds of millions of tons more GHG emissions from on-road transport than high-density urban population. These asymmetries reveal substantial per-capita inequalities between urban and rural areas, suggesting that sustainability efforts must extend beyond dense urban cores to address low-density suburban and rural settlements.

Third, intra-settlement scaling provides a micro-to-macro lens for reinterpreting city-level outcomes. City-level patterns emerge from the aggregation of cell-level processes. Urban growth operates incrementally: construction, renewal, infill, and expansion add new data points to the cell-level distribution. The characteristics of these additions, particularly population density and land-use efficiency, shape aggregate performance. In city-based scaling, expanding urban boundaries to incorporate low-density fringe areas often, though not always, dilutes sublinear effects toward near-linearity (Fig. 4b). This may also help explain why longitudinal analyses frequently observe a transition from scale economies to resource-intensive growth (Depersin & Barthelemy, 2018; Seto et al., 2011), as urban expansion typically occurs in peripheral low-density areas rather than dense cores. The policy implication is straightforward: sustainability hinges on where, and at what densities new residents are accommodated. Prioritizing infill, compact renewal, and efficient land use is central to sustainable settlement development.

## Supplementary Information

Below is the link to the electronic supplementary material.

Supplementary Information SI1. This Supplementary Information contains supplementary methodological details, additional explanations, robustness tests, figures, and results, including Supplementary Notes S1, S20, Supplementary Figures S1, S18, Supplementary Tables S1, S24, and Supplementary References. Supplementary file1 (DOCX 7278 KB)
Supplementary Information SI2.

This Supplementary Information file provides the numerical data underlying Tables S3, S4, S10, S11, S19, and S23 reported in Supplementary Information SI1.
Supplementary file2 (XLSX 47 KB)

## Data Availability

All data required to evaluate the conclusions of this study are available within the article and its Supplementary Information. The raw input data and all source code used for data processing and analysis have been deposited in a public GitHub repository (https://github.com/socialecologyboku/SociometabolicScaling) and formally archived on Zenodo (software: 10.5281/zenodo.20600537; input data: 10.5281/zenodo.20594106). Detailed documentation and instructions for utilizing the repository are available in the repository and hosted at https://socialecologyboku.github.io/SociometabolicScaling/.
