# Supplementary material for: Intra-settlement scaling across the urban–rural continuum: material stocks, service provisioning, and greenhouse gas emissions in the contiguous United States
Source: J Ind Ecol. 2026 Jul 3;30(4):1835–49. doi: 10.1007/s44498-026-00125-w (PMC13451267; doi:10.1007/s44498-026-00125-w)
Supplement: Supplementary file 1 [file 44498_2026_125_MOESM1_ESM.docx]

SUPPORTING INFORMATION FOR:

Yang, Y., Grammer, B., Prieto-Curiel, R., Frantz, D., Haberl, H. & Wiedenhofer, D. Intra-settlement scaling across the urban–rural continuum: material stocks, service provisioning, and greenhouse gas emissions in the contiguous United States. *Journal of Industrial Ecology.*

This Supplementary Information contains supplementary methodological details, additional explanations, robustness tests, figures, and results, including Supplementary Notes S1–S20, Supplementary Figures S1–S18, Supplementary Tables S1–S24, and Supplementary References.

**Note S1.** Non-universality of urban scaling laws: sensitivity to city definitions in CO₂ emissions.

In empirical studies of urban scaling, estimated scaling exponents are not universal but vary with both geographical context and the operational definition of cities. For CO₂ emissions, reported scaling regimes range from super-linear to sublinear across different studies. Table S1 and Fig. S1 compile representative examples from the literature that document these variations.

**Table S1.** CO_2_ emissions and urban population: super-linear vs. sublinear scaling.

| **Paper References** | **Response Indicators** | **Scaling relationship** | **Main findings** |
| --- | --- | --- | --- |
| Oliveira et al. (Oliveira et al., 2014) | Total CO_2_ emissions | super-linear | There is strong super-linear scaling in U.S. cities, where per capita CO_2_ emissions are substantially higher for cities with larger population sizes. |
| Fragkias et al. (Fragkias et al., 2013) | Total CO_2_ emissions | approximately linear | CO_2_ emissions of U.S. metropolitan areas scale proportionally with urban population size. |
| Rybski et al. (Rybski et al., 2016) | Total CO_2_ emissions | super-linear | Countries with lower GDP/cap values tend to exhibit scaling exponent >1 |
|  |  | sub-linear | Countries with higher GDP/cap values tend to exhibit scaling exponent <1 |
| Rao et al. (Rao et al., 2023) | Total CO_2_ emissions | sub-linear | In 308 Chinese cities, the growth rate of urban carbon emissions is substantially slower than the growth rate of urban size. |
| Hong et al. (Hong et al., 2022) | Total, industrial and residential CO_2_ emissions | sub-linear | There are sub-linear scaling relationships between urban size and total, industrial, and residential carbon emissions. |
|  | Transport CO_2_ emissions | super-linear | A super-linear relationship was identified for transportation carbon emissions. |
| Louf and Barthelemy (Louf & Barthelemy, 2014) | Transport CO_2_ emissions | super-linear | The CO_2_ emitted by cars increases super-linearly with the city’s population, primarily due to congestion. |
| Glaeser and Kahn (Glaeser & Kahn, 2010) | Transport CO_2_ emissions | sub-linear | The CO_2_ emitted by cars increases sub-linearly with the city’s population, as estimated by the distance travelled by commuters. |
| Mohajeri et al. (Mohajeri et al., 2015) | Transport CO_2_ emissions | approximately linear | Transport CO_2_ emissions scale approximately linearly with city population in Great Britain. |

**Fig. S1. Variability of urban scaling exponents for CO₂ emissions.** (a) Urban scaling regimes for CO_2_ emissions vs. population size, defined by the scaling exponent *β*: sublinear (*β* < 1), linear (*β* = 1), and super-linear (*β* > 1). (b) Regional and sectoral variation in *β* values across the USA, Europe, and Asia. Symbols represent emission sectors: total (green triangles), residential (yellow squares), industrial (orange circles), and transport (blue hexagons).

**Note S2.** Datasets.

For our analysis, we utilize open geospatial data sets encompassing population data, built environment stock data, built environment area data, building and on-road GHG emission data, and urban boundaries.

For population data, we did not adopt any publicly available population gridded data sets. Instead, we used population data from the smallest statistical unit released by the U.S. Census Bureau—census blocks population data. Census blocks represent the smallest geographic unit for which data are available. Although census block data have undergone privacy protection processes such as differential privacy algorithms and post-processing, these measures do not affect the data’s overall statistical performance (the Population Reference Bureau & the U.S. Census Bureau’s 2020 Census Data Products and Dissemination Team, n.d.). The U.S. Census Bureau conducts a comprehensive census every ten years, with the latest enumeration completed in 2020. The 2020 U.S. Census data accounts for 95.5% of all occupied housing units nationwide, with missing data in certain blocks addressed through imputation methods (Jarmin, n.d.). Therefore, this dataset is the most detailed one based on actual statistics that we could obtain. We chose not to use high-resolution population gridded data sets, such as the GHS Population Grid, because these products typically rely on proxy parameters such as building area (Schiavina, Marcello et al., 2023) or impervious surface (Swanwick et al., 2022) to estimate spatial distributions. This reliance can potentially introduce strong collinearity with the built environment area data, which serves as the primary response variable in this study, thereby compromising the validity of our results.

To process the unique population gridded data used in this study, we first downloaded census block-level population data for the United States from the United States Census Bureau website (United States Census Bureau, n.d.). This data, provided in seamless polygon shapefile format, is organized into individual documents based on each state. We then merged the 48 shapefile documents for the contiguous United States (contig. U.S.) into a single comprehensive map. In 2020, the contig. U.S. contained 8,089,668 census blocks. Within this region, the 25th percentile of census block areas is 13,176 m^2^, the median is 39,315 m^2^, and the 75th percentile is 371,996 m^2^ (Table S2). That is, half of the census blocks have diameters ranging from around 100 m to 200 m. In sparsely populated areas, block diameters may exceed 600 m, and in extremely sparsely populated regions, diameters can extend up to around 59 km. We then allocated these data to 1 km-by-1 km grids proportionally by area, thereby constructing the population base map employed in this research.

**Table S2.** Statistical description of land area, population size, and population density in contig. U.S. Census blocks.

|  | **Area (m^2^)** | **Population** | **Population density (people/km^2^)** |
| --- | --- | --- | --- |
| **Mean** | 946,262 | 41 | 1,219.76 |
| **25th Percentile** | 1,317 | 0 | 0 |
| **Median** | 39,315 | 14 | 116.925 |
| **75th Percentile** | 371,996 | 44 | 1,589.948 |
| **Maximum** | 3,490,105,000 | 8,727 | 3,000,000.000 |

For material stock data, we downloaded the Material Stock Map of the CONUS (version V.1.1) from the Zenodo repository (Frantz et al., 2023). This dataset, based on high-resolution Earth Observation data (Sentinel-1 and Sentinel-2), provides a detailed 10 m gridded map of the area, material stock mass of mobility infrastructure, and the footprint, volume, and material stock mass of buildings in the contig. U.S. Each category, such as building types, is further divided into subcategories. The data were split into seven regions and uploaded into sub-repositories. We derived the material mass of buildings and mobility infrastructure, including streets, by merging and summing the subcategory data from these seven regions. For example, we combined the “single-family,” “multifamily,” and “lightweight” categories into the “residential building” category for our study. This aggregated data was then resampled to a 1 km resolution for analysis. Similarly, the area of mobility infrastructures was derived from the source data. The floor area of buildings was calculated using the footprint and volume information, assuming a floor-to-floor height of 3 m for residential buildings and 3.6 m for non-residential buildings.

Notably, for building indicators, building mass and building floor area were generated through different methodological pathways. According to the source data methodology of Frantz et al. (2023), building mass was estimated by first predicting building type and height from Earth Observation data. Vector-based building footprints were converted into raster-based measures of building footprint area at 10 m spatial resolution. Building footprint area [m²] and building height [m] were then multiplied to estimate aboveground building volume [m³], which was subsequently multiplied by mass-per-volume factors [t m⁻³] that vary by building type and climate zone, yielding material-specific building mass [t]. Thus, building mass is based on building volume, building type, and climate-zone-specific coefficients.

In this study, building floor area was calculated as building footprint multiplied by the estimated number of floors. The number of floors was estimated by dividing building height by assumed average floor heights, namely 3.0 m for residential buildings and 3.6 m for non-residential buildings. Therefore, although building floor area and building mass are closely related, they are not directly derived from one another and should not be treated as interchangeable indicators.

The same logic applies to total infrastructure/street area and mass. According to Frantz et al. (2023), infrastructure area [m²] for each mobility infrastructure type was multiplied by mass-per-area factors [t m⁻²] that vary by mobility infrastructure type, material, and climate zone, yielding infrastructure mass [t] at 10 m spatial resolution. Therefore, infrastructure mass is not obtained by multiplying total infrastructure area by a single constant. If this were the case, the two indicators would be mathematically equivalent and would necessarily produce identical scaling results. Instead, the relationship between infrastructure area and infrastructure mass depends on additional information, including infrastructure type, material composition, and climate-zone-specific factors.

We retained these related indicators for two main reasons. First, each indicator carries a distinct conceptual meaning. Building footprint represents land occupation, building floor area reflects usable built space and service provision, building mass represents material stock. In previous urban scaling studies, building footprint, building floor area, and street area have been used depending on data availability and research focus. Including these indicators therefore improves comparability with existing studies and makes the results relevant to different research communities. Second, these correlated indicators are not simple multipliers of one another. Their relationships are shaped by multiple factors, including building or infrastructure type, material composition, and climate zone. Therefore, the correlation among indicators does not affect the validity of each population–indicator scaling relationship analyzed in this study.

GHG emissions data for this study were derived from two primary sources. Road transportation-related emissions were obtained from the DARTE dataset (Gately et al., 2019, pp. 1980–2017), while emissions from buildings and industry were sourced from the Vulcan dataset (Gurney et al., 2020). Both datasets were accessed through the Earth Science Data Systems (ESDS) Program website.

The DARTE dataset provides a 38-year inventory of annual on-road CO_2_ emissions for the conterminous United States at a 1 km resolution, and for this study, we utilized the 2017 data. DARTE employs a bottom-up approach by combining roadway-level traffic data with state-specific emission factors for different vehicle types on both urban and rural roads. The primary data source is the Highway Performance Monitoring System (HPMS), which tracks vehicle miles travelled (VMT).

The Vulcan dataset offers annual gridded emissions data at a 1 km resolution for the period 2010-2015, and we employed the 2015 data to represent residential and non-residential emissions. The residential sector includes emissions from household fossil fuel use, such as natural gas and heating oil. The commercial sector encompasses emissions from office and retail buildings using fossil fuels for heating and operations. The industrial sector includes emissions from manufacturing facilities and factories using fossil fuels in production. The data were sourced from authoritative references such as the U.S. Environmental Protection Agency’s National Emissions Inventory and the Energy Information Administration. The DARTE and Vulcan datasets use coordinate systems that differ from that of the mass dataset; therefore, we utilized the coordinate system of the mass dataset to ensure consistency across the maps from these various sources.

This study involves four city boundaries. The first boundary, the GHS Urban Centre Database (GHS-UCDB), was downloaded from the European Commission’s website (Florczyk et al., 2019). The GHS-UCDB defines “urban centres” based on specific thresholds for resident population and built-up surface area within a 1 km-by-1 km global grid. The second boundary is from the U.S. Census Bureau’s Urban Areas Dataset (USCB-UADS) 2023, downloaded from the U.S. Department of Transportation’s website (the United States Census Bureau & the U.S. Department of Transportation, 2024). This dataset delineates areas of high population density and urban land use, providing a comprehensive representation of the urban footprint. It includes 2,645 urban areas with a minimum population of 5,000 or at least 2,000 housing units. The third boundary is Morphological Urban Areas (MUAs) (Taubenböck et al., 2019). MUAs are identified based on settlement area, settlement area proportion and density within the settlements, distinguishing urban areas from surrounding rural or low-density regions. The fourth is the GHSL-OECD Functional Urban Areas (GHS-FUA), defined jointly by the OECD and the European Union (CEU. JRC., 2019; Schiavina et al., 2019). GHS-FUAs consist of contiguous local administrative units composed of a city and its commuting zone, where at least 15% of the working population commute to the core city.

**Note S3.** Justification and sensitivity of assumed average floor heights.

The assumed average floor heights, 3.0 m for residential buildings and 3.6 m for non-residential buildings, are broadly consistent with the Prototype Building Models and related commercial reference building models developed by the Pacific Northwest National Laboratory (PNNL) for the U.S. Department of Energy (DOE). These models are widely used in building energy simulation, energy code analysis, and building stock studies in the United States.

For residential buildings, the DOE/PNNL prototype models commonly use a floor-to-floor height of approximately 10 ft, equivalent to about 3.05 m, for representative residential buildings. This height accounts for a standard 9-ft clear ceiling height plus approximately 1 ft for structural joists and floor assemblies (Mendon & Taylor, 2014; PNNL, 2023). Therefore, we used 3.0 m as a reasonable approximation for residential buildings.

For non-residential buildings, DOE commercial prototype and reference buildings commonly use floor-to-floor heights in the range of approximately 10–13 ft, depending on building type and floor function. A value of 3.6 m, approximately 11.8 ft, represents a mid-range estimate that accommodates the necessary plenum space, typically 2–4 ft, required for HVAC ductwork, fire protection, and electrical systems in standard commercial settings (Deru et al., 2011).

We note that the non-residential category also includes industrial buildings, whose typical floor-to-floor heights may be higher, for example around 4.2 m or more depending on building type and use. Therefore, the assumed 3.6 m height may underestimate the floor height for some industrial buildings. However, in our procedure, the estimated number of floors was rounded down to the nearest integer, which reduces the sensitivity of estimated floor counts to small variations in assumed floor height.

Regarding sensitivity, changing the assumed floor height would rescale the estimated gross floor area within each building-use category. In the log-log scaling model, such a multiplicative change mainly affects the intercept, or scaling prefactor, rather than the scaling exponent, as long as the same assumption is applied consistently within each category. Therefore, the assumed floor heights may influence the absolute magnitude of estimated gross floor area, but are unlikely to substantially affect the estimated scaling exponents or the main conclusions of the study.

**Note S4.** Comparisons of results using total amount vs. density values in a grid-based scaling model.

The distinction between using implied density measures (i.e., total value as a proxy for “density”) or actual density measures (i.e., total value divided by grid area) when using a regular grid instead of cities as units of observation has no impact on the scaling coefficients. To see this, let $P_{i}$ be the population of grid cell *i*, $D_{i}$ its density, and $A_{i}$ its area (so $P_{i}=A_{i}{\cdot D}_{i}$). Then, we express the variable of interest $Y_{i}$ as:

$Y_{i}=\alpha P_{i}^{\beta}=\alpha(A_{i}D_{i})^{\beta}$,

(1)

for the corresponding coefficients $\alpha$ and $\beta$. Then, since we are using a regular grid, the area of each cell can be expressed a constant:

$A_{i}=\nu$,

(2)

for some $\nu$ > 0. Then, the scaling expression becomes:

$Y_{i}=(\alpha\nu^{\beta})D_{i}^{\beta}=\alpha^{'}D_{i}^{\beta}$,

(3)

for some value of $\alpha^{'}$ which does not depend on the grid cell, and for the same $\beta$ as the one obtained for the total population. Therefore, when using a regular grid, the scaling coefficient of interest, $\beta$, is mathematically the same whether the analysis is based on total population or density. Specifically, in the context of this study with a 1 km-by-1km grid cell, the results of both analyses are the same.

Table S3 presents a specific example using 20 km-by-20 km grid cells as the observation unit, where scaling results are reported based on two explanatory variables: total population within the grid (unit: people) and population density (unit: people/km²). To ensure comparability between the two approaches, no variable filters were applied. The scaling exponent (*β*) and goodness of fit (*R*^2^) remain identical, with only the factor *α*′ differing.

**Table S3.** Comparison of scaling results using total population vs. population density in 20 km-by-20 km grids.

| **Variables** | Total population as explorational variable | | | Population density as explorational variable | | |
| --- | --- | --- | --- | --- | --- | --- |
|  | $\beta$ | $\alpha^{'}$ | $R^{2}$ | $\beta$ | $\alpha^{'}$ | $R^{2}$ |
| Total mass | 0.43 | **11.68** | 0.77 | 0.43 | **14.25** | 0.77 |
| Total building mass | 0.88 | **6.48** | 0.89 | 0.88 | **11.74** | 0.89 |
| Residential building mass | 0.92 | **5.73** | 0.89 | 0.92 | **11.27** | 0.89 |
| Non-residential building mass | 0.86 | **5.10** | 0.78 | 0.86 | **10.25** | 0.78 |
| Total mobility infrastructure mass | 0.35 | **11.94** | 0.72 | 0.35 | **14.02** | 0.72 |
| Street mass | 0.32 | **11.98** | 0.68 | 0.32 | **13.87** | 0.68 |
| Total building footprint | 0.83 | **6.45** | 0.90 | 0.83 | **11.41** | 0.90 |
| Residential building footprint | 0.87 | **5.83** | 0.90 | 0.87 | **11.04** | 0.90 |
| Non-residential building footprint | 0.87 | **5.83** | 0.90 | 0.87 | **11.04** | 0.90 |
| Total building floor area | 0.90 | **6.09** | 0.89 | 0.90 | **11.50** | 0.89 |
| Residential building floor area | 0.95 | **5.42** | 0.89 | 0.95 | **11.09** | 0.89 |
| Non-residential building floor area | 0.88 | **4.65** | 0.78 | 0.88 | **9.90** | 0.78 |
| Total mobility infrastructure area | 0.28 | **13.25** | 0.68 | 0.28 | **14.96** | 0.68 |
| Street area | 0.25 | **13.33** | 0.64 | 0.25 | **14.85** | 0.64 |
| Residential buildings emissions | 0.63 | **1.86** | 0.77 | 0.63 | **5.63** | 0.77 |
| Commercial buildings emissions | 0.66 | **0.69** | 0.71 | 0.66 | **4.64** | 0.71 |
| Commercial and industrial buildings emissions | 0.74 | **0.91** | 0.65 | 0.74 | **5.33** | 0.65 |
| On-road emissions | 0.59 | **12.10** | 0.70 | 0.59 | **15.64** | 0.70 |

That is, when comparing data across regular grids, whether using ‘total’ values, density values, or different variable units within the grid, only the coefficient value of *α*' is affected, the scaling exponent $\beta$ remains unchanged.

**Note S5.** Sensitivity Analysis on Grid Alignment (Grid Shifting).

In gridded spatial analyses, the placement of grid boundaries, such as the location of the grid origin, is arbitrary. As a result, the specific grid alignment used may introduce uncertainties into the analysis. To quantify the sensitivity of regression results to grid alignment, we conducted a grid shifting analysis, by systematically shifting the grid origin in 1 km steps to generate alternative alignments. This approach allows us to evaluate how grid shifts impact regression results, particularly the estimated scaling exponents (*β*) and goodness of fit (*R*²).

Using 1 km-by-1 km input maps as the base resolution, we aggregated the data to target resolutions of 5 km, 10 km, and 30 km. For each target resolution, we applied systematic grid shifts in all possible directions. Specifically, the number of grid shift variants is determined by $(\text{target resolution}/\text{base resolution})^{2}$. For example, when the target resolution is 30 km and the base resolution is 1 km, this results in $(30/1)^{2}=900$ grid shift variants, representing all possible 1 km-shifted alignments within a 30×30 km window.

For each resolution and grid shift variant, we performed log–log linear regressions between population and various infrastructure-related variables. We then computed the mean, standard deviation, maximum, and minimum values of the regression results (*β* and *R*²) across all shift variants (Table S4). We found that the standard deviation of both *β* and *R*² tends to increase as the target resolution becomes coarser, which may reflect the accumulation of alignment-related errors. Nevertheless, even at the 30 km resolution, the variation remains minimal, with standard deviations below 0.004 for *β* and 0.006 for *R*², indicating highly robust scaling relationships.

In conclusion, the results demonstrate that grid shifts introduce only negligible deviations in regression outcomes. Both the *β* and *R*² remain highly stable across all grid-aligned variants, confirming the robustness of our findings against uncertainties related to grid placement.

**Table S4.** Sensitivity of *β* and *R*² to Grid Alignment Across Different Target Resolutions.

| **Target resolution** | **Variables** | ***β*** | | | | ***R*^2^** | | | |
| --- | --- | --- | --- | --- | --- | --- | --- | --- | --- |
|  |  | mean | std | max | min | mean | std | max | min |
| 5km | Total mobility infrastructure area | 0.3261 | 0.0004 | 0.3269 | 0.3253 | 0.5963 | 0.0014 | 0.5985 | 0.5930 |
|  | Total mobility infrastructure mass | 0.3924 | 0.0004 | 0.3931 | 0.3918 | 0.5732 | 0.0009 | 0.5748 | 0.5717 |
|  | Non-residential building floor area | 0.8225 | 0.0008 | 0.8238 | 0.8206 | 0.5485 | 0.0009 | 0.5499 | 0.5460 |
|  | Non-residential building footprint | 0.7349 | 0.0007 | 0.7362 | 0.7332 | 0.5322 | 0.0010 | 0.5335 | 0.5299 |
|  | Non-residential building mass | 0.8185 | 0.0008 | 0.8198 | 0.8166 | 0.5537 | 0.0010 | 0.5553 | 0.5514 |
|  | Commercial and industrial buildings emissions | 0.7120 | 0.0005 | 0.7127 | 0.7110 | 0.5644 | 0.0005 | 0.5652 | 0.5632 |
|  | Commercial buildings emissions | 0.6610 | 0.0005 | 0.6617 | 0.6601 | 0.6097 | 0.0003 | 0.6102 | 0.6091 |
|  | On-road emissions | 0.6808 | 0.0004 | 0.6817 | 0.6803 | 0.5804 | 0.0007 | 0.5818 | 0.5793 |
|  | Residential building floor area | 0.9209 | 0.0006 | 0.9221 | 0.9199 | 0.8272 | 0.0006 | 0.8283 | 0.8260 |
|  | Residential building footprint | 0.8549 | 0.0005 | 0.8561 | 0.8539 | 0.8316 | 0.0007 | 0.8332 | 0.8303 |
|  | Residential building mass | 0.9124 | 0.0006 | 0.9138 | 0.9113 | 0.8074 | 0.0007 | 0.8089 | 0.8060 |
|  | Residential buildings emissions | 0.6611 | 0.0003 | 0.6617 | 0.6604 | 0.7162 | 0.0004 | 0.7168 | 0.7155 |
|  | Street area | 0.2864 | 0.0003 | 0.2871 | 0.2858 | 0.5657 | 0.0015 | 0.5681 | 0.5622 |
|  | Street mass | 0.3468 | 0.0003 | 0.3475 | 0.3463 | 0.5399 | 0.0009 | 0.5420 | 0.5384 |
|  | Total building floor area | 0.9272 | 0.0006 | 0.9284 | 0.9262 | 0.8234 | 0.0006 | 0.8250 | 0.8222 |
|  | Total building footprint | 0.8562 | 0.0005 | 0.8573 | 0.8553 | 0.8313 | 0.0007 | 0.8330 | 0.8298 |
|  | Total building mass | 0.9191 | 0.0006 | 0.9203 | 0.9181 | 0.8091 | 0.0008 | 0.8110 | 0.8076 |
|  | Total mass | 0.5056 | 0.0004 | 0.5063 | 0.5047 | 0.6815 | 0.0008 | 0.6831 | 0.6800 |
| 10km | Total mobility infrastructure area | 0.3065 | 0.0005 | 0.3074 | 0.3051 | 0.6577 | 0.0025 | 0.6639 | 0.6527 |
|  | Total mobility infrastructure mass | 0.3739 | 0.0007 | 0.3757 | 0.3725 | 0.6570 | 0.0024 | 0.6624 | 0.6517 |
|  | Non-residential building floor area | 0.8998 | 0.0014 | 0.9038 | 0.8965 | 0.7027 | 0.0011 | 0.7054 | 0.7001 |
|  | Non-residential building footprint | 0.8025 | 0.0013 | 0.8057 | 0.7998 | 0.6888 | 0.0009 | 0.6912 | 0.6865 |
|  | Non-residential building mass | 0.8889 | 0.0014 | 0.8922 | 0.8857 | 0.7057 | 0.0010 | 0.7083 | 0.7033 |
|  | Commercial and industrial buildings emissions | 0.7656 | 0.0009 | 0.7677 | 0.7636 | 0.6453 | 0.0015 | 0.6479 | 0.6418 |
|  | Commercial buildings emissions | 0.6888 | 0.0008 | 0.6906 | 0.6872 | 0.6861 | 0.0008 | 0.6883 | 0.6844 |
|  | On-road missions | 0.6528 | 0.0008 | 0.6549 | 0.6503 | 0.6565 | 0.0010 | 0.6589 | 0.6545 |
|  | Residential building floor area | 0.9601 | 0.0009 | 0.9621 | 0.9578 | 0.8692 | 0.0007 | 0.8712 | 0.8678 |
|  | Residential building footprint | 0.8889 | 0.0008 | 0.8912 | 0.8866 | 0.8738 | 0.0007 | 0.8760 | 0.8721 |
|  | Residential building mass | 0.9488 | 0.0009 | 0.9510 | 0.9462 | 0.8564 | 0.0006 | 0.8583 | 0.8548 |
|  | Residential buildings emissions | 0.6659 | 0.0006 | 0.6677 | 0.6646 | 0.7619 | 0.0007 | 0.7634 | 0.7602 |
|  | Street area | 0.2702 | 0.0005 | 0.2711 | 0.2688 | 0.6200 | 0.0028 | 0.6270 | 0.6137 |
|  | Street mass | 0.3328 | 0.0006 | 0.3343 | 0.3314 | 0.6200 | 0.0025 | 0.6263 | 0.6142 |
|  | Total building floor area | 0.9564 | 0.0012 | 0.9594 | 0.9539 | 0.8731 | 0.0008 | 0.8755 | 0.8716 |
|  | Total building footprint | 0.8793 | 0.0010 | 0.8815 | 0.8768 | 0.8826 | 0.0007 | 0.8839 | 0.8808 |
|  | Total building mass | 0.9415 | 0.0010 | 0.9438 | 0.9386 | 0.8675 | 0.0007 | 0.8690 | 0.8656 |
|  | Total mass | 0.4742 | 0.0008 | 0.4759 | 0.4725 | 0.7393 | 0.0020 | 0.7434 | 0.7343 |
| 30km | Total mobility infrastructure area | 0.3071 | 0.0023 | 0.3144 | 0.2990 | 0.7024 | 0.0059 | 0.7199 | 0.6831 |
|  | Total mobility infrastructure mass | 0.3687 | 0.0022 | 0.3755 | 0.3605 | 0.7495 | 0.0059 | 0.7638 | 0.7311 |
|  | Non-residential building floor area | 0.9190 | 0.0032 | 0.9276 | 0.9104 | 0.8416 | 0.0027 | 0.8499 | 0.8354 |
|  | Non-residential building footprint | 0.8157 | 0.0034 | 0.8243 | 0.8070 | 0.8304 | 0.0028 | 0.8401 | 0.8227 |
|  | Non-residential building mass | 0.8941 | 0.0038 | 0.9045 | 0.8842 | 0.8398 | 0.0027 | 0.8483 | 0.8330 |
|  | Commercial and industrial buildings emissions | 0.8487 | 0.0037 | 0.8579 | 0.8403 | 0.7040 | 0.0041 | 0.7150 | 0.6938 |
|  | Commercial buildings emissions | 0.7548 | 0.0033 | 0.7632 | 0.7440 | 0.7833 | 0.0028 | 0.7902 | 0.7748 |
|  | On-road emissions | 0.6406 | 0.0031 | 0.6503 | 0.6309 | 0.7578 | 0.0035 | 0.7667 | 0.7475 |
|  | Residential building floor area | 0.9819 | 0.0024 | 0.9887 | 0.9754 | 0.9158 | 0.0016 | 0.9199 | 0.9107 |
|  | Residential building footprint | 0.8995 | 0.0020 | 0.9054 | 0.8947 | 0.9218 | 0.0018 | 0.9270 | 0.9149 |
|  | Residential building mass | 0.9545 | 0.0023 | 0.9601 | 0.9487 | 0.9064 | 0.0017 | 0.9119 | 0.9006 |
|  | Residential buildings emissions | 0.7053 | 0.0025 | 0.7107 | 0.6971 | 0.8184 | 0.0029 | 0.8242 | 0.8106 |
|  | Street area | 0.2725 | 0.0024 | 0.2799 | 0.2641 | 0.6452 | 0.0061 | 0.6629 | 0.6269 |
|  | Street mass | 0.3312 | 0.0025 | 0.3390 | 0.3223 | 0.6994 | 0.0063 | 0.7146 | 0.6810 |
|  | Total building floor area | 0.9319 | 0.0025 | 0.9398 | 0.9247 | 0.9277 | 0.0018 | 0.9324 | 0.9218 |
|  | Total building footprint | 0.8476 | 0.0025 | 0.8556 | 0.8407 | 0.9402 | 0.0017 | 0.9458 | 0.9342 |
|  | Total building mass | 0.8981 | 0.0029 | 0.9069 | 0.8903 | 0.9282 | 0.0017 | 0.9337 | 0.9222 |
|  | Total mass | 0.4572 | 0.0022 | 0.4633 | 0.4509 | 0.8153 | 0.0038 | 0.8260 | 0.8041 |

**Note S6.** Sensitivity Analysis on the Definition of “Residential”.

Our current calculation of grid-level residential floor area and building mass excludes the potential residential functions embedded within high-rise and skyscraper categories in the original datasets. To assess the sensitivity of our results to the functional definition of residential buildings, we conducted an uncertainty analysis by reclassifying a portion of the floor area and mass of high-rise and skyscraper buildings as “residential”.

Due to the lack of consistent nationwide data on building functions at the grid level, and considering that the function of high-rise buildings often varies between urban cores and peripheral areas, we adopted a rule-based estimation approach (Table S5). Specifically, we estimated residential shares for high-rise and skyscraper buildings based on the urban–rural classification from the GHS-SMOD dataset. Each grid cell was assigned a residential share according to its SMOD class, and this share was uniformly applied to both high-rise and skyscraper structures. Although skyscrapers may in practice contain slightly less residential floor area than high-rises, we treated them equally in this analysis to reflect a maximal assumption of potential residential use and to test the upper-bound impact of such reclassification on our results.

**Table S5.** Residential Allocation Rules for High-Rise and Skyscraper Buildings Based on SMOD Classification

| **Simplified Class** | **Included SMOD Classes** | **Region Description** | **Residential Share in High-rise** | **Residential Share in Skyscraper** |
| --- | --- | --- | --- | --- |
| **Urban centre** | 30 | Urban Centre | 50% | 50% |
| **Suburban** | 23, 22, 21 | Dense, Semi-dense, Suburban | 70% | 70% |
| **Rural area** | 11,12,13 | Rural settlements | 90% | 90% |

We integrate the estimated residential share of high-rise and skyscraper buildings into the residential building category and subtract the corresponding portion from the non-residential category to ensure that the total floor area and mass remain unchanged. As a result, four variables are updated: residential floor area, residential building mass, non-residential floor area, and non-residential building mass. We then re-run the regression analysis across the entire contiguous United States and compare the changes in the scaling exponent *β* and the goodness of fit *R*², to evaluate the sensitivity of our findings to this functional reclassification (Table S6 and S7).

The comparison reveals that at the 1 km-by-1 km grid level, incorporating residential functions within high-rise and skyscraper buildings results in only negligible changes in both the estimated scaling exponent (*β*) and model fit (*R*²) for residential and non-residential categories. This indicates that our original classification method, despite adopting a simplified definition of residential use, yields robust and reliable results.

**Table S6.** Regression Results After Reclassifying High-Rise and Skyscraper Buildings with Assumed Residential Shares

| **Variables** | ***β*** | **Intercept** | ***R*^2^** | **Gird size** | **Population filter** |
| --- | --- | --- | --- | --- | --- |
| Residential building floor area | 0.824 | 5.577 | 0.622 | 1km | x = 1 |
|  | 0.911 | 5.208 | 0.638 | 1km | x = 5 |
|  | 0.932 | 5.106 | 0.647 | 1km | x = 10 |
| Non-residential building floor area | 0.607 | 4.710 | 0.280 | 1km | x = 1 |
|  | 0.754 | 4.000 | 0.308 | 1km | x = 5 |
|  | 0.842 | 3.52 | 0.316 | 1km | x = 10 |
| Residential building mass | 0.814 | 5.711 | 0.597 | 1km | x <= 1 |
|  | 0.892 | 5.383 | 0.610 | 1km | x <= 5 |
|  | 0.907 | 5.310 | 0.618 | 1km | x <= 10 |
| Non-residential building mass | 0.604 | 4.989 | 0.276 | 1km | x <= 1 |
|  | 0.745 | 4.306 | 0.302 | 1km | x <= 5 |
|  | 0.830 | 3.848 | 0.310 | 1km | x <= 10 |

**Table S7**: Baseline Regression Results Using the Original Functional Classification

| **Variables** | ***β*** | **Intercept** | ***R*^2^** | **Gird size** | **Population filter** |
| --- | --- | --- | --- | --- | --- |
| Residential building floor area | 0.823 | 5.579 | 0.621 | 1km | x <= 1 |
|  | 0.910 | 5.212 | 0.637 | 1km | x <= 5 |
|  | 0.930 | 5.112 | 0.646 | 1km | x <= 10 |
| Non-residential building floor area | 0.608 | 4.710 | 0.280 | 1km | x <= 1 |
|  | 0.754 | 4.000 | 0.308 | 1km | x <= 5 |
|  | 0.842 | 3.521 | 0.316 | 1km | x <= 10 |
| Residential building mass | 0.824 | 5.577 | 0.622 | 1km | x = 1 |
|  | 0.911 | 5.208 | 0.638 | 1km | x = 5 |
|  | 0.932 | 5.106 | 0.647 | 1km | x = 10 |
| Non-residential building mass | 0.608 | 4.710 | 0.280 | 1km | x = 1 |
|  | 0.754 | 3.998 | 0.308 | 1km | x = 5 |
|  | 0.842 | 3.523 | 0.316 | 1km | x = 10 |

**Notes S7.** Comparison between OLS Regression and Spatial Regression Model.

In conventional urban scaling law analyses, the linear regression model is typically estimated using ordinary least squares (OLS) in log-log space without explicitly accounting for spatial relationships between cities. One difference between grid-based regression and city-based regression is that gridded data inherently contain richer spatial adjacency information. To systematically assess whether spatial proximity among grid cells introduces additional uncertainty or reduces the reliability of OLS estimates, we followed a three-step procedure: 1) Residual Spatial Autocorrelation Analysis. We examined whether the residuals from the OLS regression used in this study exhibit spatial autocorrelation. 2) Spatial Model Comparison. We introduced two alternative spatial regression models and compared their regression results (*β* and *R*²) with those from the OLS model that ignores spatial dependence. 3) Conclusion. We evaluated whether accounting for spatial dependence materially changes the estimated scaling relationships.

**Residual Spatial Autocorrelation Check**

First, we tested whether the regression residuals from the OLS model in our study exhibit spatial autocorrelation. Spatial autocorrelation was quantified at Global scale using Moran’s I.

The analysis was conducted at multiple spatial resolutions (1000 m, 5000 m, …, 30000 m). For each resolution: (a) we ran an OLS regression in log–log space (with population values filtered to ≤1), (b) computed the residuals, and (c) tested for spatial autocorrelation using Moran’s I.

Results indicate that (Fig. S2), across most variables, Moran’s I values are lowest at the finest resolution (1 km-by-1km), where they typically range from 0.13 to 0.31 for all variables except those related to emissions. Moran’s I increases with spatial aggregation and peaks at intermediate resolutions (approximately 10–20 km), before declining again at coarser scales. This pattern suggests that for many infrastructure-related variables, spatial autocorrelation in OLS residuals is most pronounced at larger scales, where local heterogeneity is smoothed out. In contrast, carbon-emission variables display a distinct trend: their Moran’s I is highest at the finest resolution (up to 0.75) and decreases steadily as resolution becomes coarser. This likely reflects the way the emission data were constructed. For all variables and resolutions, the associated p-values are effectively zero (p < 1 × 10⁻⁶), indicating that all observed spatial clustering patterns are highly significant.

These findings suggest that OLS residuals exhibit statistically significant positive spatial autocorrelation across all scales and variables. However, at the 1 km resolution, spatial clustering is relatively weak for most variables, raising the question of whether such autocorrelation substantially affects the reliability of OLS estimates. This is the key issue we aim to understand. To address this, further analysis using spatial regression techniques (spatial lag and error models) was conducted to assess the robustness of the results.

**Fig. S2.** Spatial Autocorrelation of OLS Residuals Across Scales.

**Spatial Models analysis: Comparing OLS, Spatial Error Models and Spatial Lag Models**

Given the presence of spatial autocorrelation, we were wondering whether integrating spatial correlation into the model (using spatial lag and error models) could influence the scaling results, and perhaps also help reduce the spatial autocorrelation in the residuals. So, we tested Spatial Error Model (SEM) and Spatial Lag Model (SLM) and compared the results from all these different kinds of models (OLS, SEM, SLM). Table S8 shows the model equations for each approach. Specifically, SLM incorporates spatial dependence in the dependent variable, capturing potential spillover effects where the outcome in one unit is directly influenced by neighbouring outcomes. SEM attributes spatial dependence to unobserved spatially correlated factors, assuming that the main spatial effect operates through the error structure rather than direct influence between units.

**Table S8.** Mathematical specifications of the OLS, Spatial Error Model (SEM), and Spatial Lag Model (SLM).

| **Models** | **Equations** | **Parameter explanations** |
| --- | --- | --- |
| **OLS** | $\log Y_{i}=\alpha+\beta\log N_{i}+\epsilon_{i}$, | where $Y_{i}$ is the outcome variable of interest (e.g., infrastructure mass, emissions) in unit $i$, $N_{i}$ is the population of unit $i$, $\beta$ is the scaling exponent in log–log space, $\epsilon_{i}$ is the error term. |
| **SLM**  (spatial lag model) | $\log Y_{i}=\alpha+\beta\log N_{i}+\rho\sum_{j} w_{ij}\log Y_{j}+\epsilon_{i}$, | where $w_{ij}$ represents the spatial weight between units $i$ and $j$ (our test based on Queen contiguity), $\sum_{j} w_{ij}logY_{j}$ is the spatial lag of the dependent variable, $\rho$ is the spatial lag coefficient, measuring the influence of neighbouring units’ outcomes on unit $i$. |
| **SEM**  (spatial error model) | $\log Y_{i}=\alpha+\beta\log N_{i}+\epsilon_{i}$,  $\epsilon_{i}= \lambda\sum_{j} w_{ij}\epsilon_{i}+u_{i}$, | where $\epsilon_{i}$ is the spatially correlated error term, $w_{ij}$ is the spatial weights matrix, $\lambda$ is the spatial error coefficient, capturing the degree of autocorrelation in residuals, $u_{i}$ is an i.i.d. error term. |

We ran the models mentioned above at multiple resolutions (1000 m, 5000 m, 10000 m, 20000 m, 30000 m) for three selected variables—total mass, total building mass, and residential building mass—which were among the top-ranked variables in the residual spatial autocorrelation analysis. As shown in Table S9 and Fig. S3, our main findings are:

(1) The OLS estimations were implemented using both the “spreg” module in PySAL, which is designed for spatial econometric analysis, and the “scipy.stats” module, a general-purpose statistical library in Python (used in the main text). The results from “spreg” were identical to those obtained with “scipy”, confirming that the estimated coefficients and fit measures are robust to the choice of statistical package for OLS estimation.

(2) The SLM yields modest improvements in model fit compared to OLS, increasing R² by at most 0.04. At the 1 km resolution, the estimated spatial lag coefficient (spillover effect) is relatively most pronounced. However, by the 5 km resolution, the lag effect disappears. More importantly, residual spatial autocorrelation remains largely unchanged between SLM and OLS, indicating that the detected spatial dependence is not primarily the result of direct spillovers (i.e., a unit’s value being strongly determined by neighbouring units).

(3) In contrast, the SEM consistently removes residual spatial autocorrelation across all tested scales, suggesting that the dependence observed in the OLS residuals is linked to underlying spatial structures rather than inter-unit interactions. Despite this correction, SEM and OLS produce very similar scaling exponents (*β*), with SEM estimates typically slightly lower; the difference $\Delta\beta=\beta_{\text{SEM}}-\beta_{\text{OLS}}$ lies within [0, 0.07] across all scales, and *R*² values are effectively identical. These small differences indicate that *β* is robust to spatial error correction, and that the main scaling relationships reported in the paper are not substantially biased by unmodeled spatial dependence.

As a conclusion of the analysis, we find that although spatial autocorrelation is present in the residuals, it does not substantially affect the estimated *β* or *R*². This indicates that the unmodeled spatial dependence in the main text is unlikely to introduce substantial bias into the results or their interpretation.

**Table S9.** Comparison of OLS, SLM, and SEM estimates for selected variables.

| **Grid size** | **Variables** |  | **method** | | |
| --- | --- | --- | --- | --- | --- |
|  |  |  | OLS | SLM | SEM |
| 1000m | Total mass | *β* | 0.63 | 0.53 | 0.60 |
|  |  | *R*^2^ | 0.458 | 0.496 | 0.458 |
|  |  | Morans’I | 0.235^***^ | 0.236^***^ | -0.014^***^ |
|  | Total building mass | *β* | 0.84 | 0.75 | 0.82 |
|  |  | *R*^2^ | 0.583 | 0.604 | 0.583 |
|  |  | Morans’I | 0.211^***^ | 0.211^***^ | -0.020^***^ |
|  | Residential building mass | *β* | 0.81 | 0.81 | 0.81 |
|  |  | *R*^2^ | 0.596 | 0.596 | 0.596 |
|  |  | Morans’I | 0.211^***^ | 0.211^***^ | -0.023^***^ |
| 5000m | Total mass | *β* | 0.51 | 0.50 | 0.50 |
|  |  | *R*^2^ | 0.682 | 0.686 | 0.682 |
|  |  | Morans’I | 0.383^***^ | 0.384^***^ | -0.024^***^ |
|  | Total building mass | *β* | 0.92 | 0.91 | 0.91 |
|  |  | *R*^2^ | 0.809 | 0.812 | 0.809 |
|  |  | Morans’I | 0.360^***^ | 0.361^***^ | -0.041^***^ |
|  | Residential building mass | *β* | 0.91 | 0.91 | 0.91 |
|  |  | *R*^2^ | 0.808 | 0.809 | 0.808 |
|  |  | Morans’I | 0.447^***^ | 0.448^***^ | -0.048^***^ |
| 10000m | Total mass | *β* | 0.47 | 0.47 | 0.47 |
|  |  | *R*^2^ | 0.741 | 0.744 | 0.741 |
|  |  | Morans’I | 0.399^***^ | 0.400^***^ | -0.023^***^ |
|  | Total building mass | *β* | 0.94 | 0.92 | 0.93 |
|  |  | *R*^2^ | 0.867 | 0.870 | 0.867 |
|  |  | Morans’I | 0.367^***^ | 0.368^***^ | -0.040^***^ |
|  | Residential building mass | *β* | 0.95 | 0.91 | 0.92 |
|  |  | *R*^2^ | 0.857 | 0.866 | 0.857 |
|  |  | Morans’I | 0.504^***^ | 0.506^***^ | -0.044^***^ |
| 20000m | Total mass | *β* | 0.45 | 0.44 | 0.44 |
|  |  | *R*^2^ | 0.795 | 0.803 | 0.795 |
|  |  | Morans’I | 0.375^***^ | 0.377^***^ | -0.014^***^ |
|  | Total building mass | *β* | 0.92 | 0.89 | 0.90 |
|  |  | *R*^2^ | 0.912 | 0.916 | 0.912 |
|  |  | Morans’I | 0.346^***^ | 0.345^***^ | -0.030^***^ |
|  | Residential building mass | *β* | 0.96 | 0.88 | 0.90 |
|  |  | *R*^2^ | 0.894 | 0.908 | 0.894 |
|  |  | Morans’I | 0.551^***^ | 0.552^***^ | -0.033^***^ |
| 30000m | Total mass | *β* | 0.45 | 0.43 | 0.43 |
|  |  | *R*^2^ | 0.816 | 0.829 | 0.816 |
|  |  | Morans'I | 0.352^***^ | 0.356^***^ | -0.008 |
|  | Total building mass | *β* | 0.90 | 0.84 | 0.87 |
|  |  | *R*^2^ | 0.928 | 0.933 | 0.928 |
|  |  | Morans’I | 0.310^***^ | 0.307^***^ | -0.017^*^ |
|  | Residential building mass | *β* | 0.95 | 0.86 | 0.88 |
|  |  | *R*^2^ | 0.907 | 0.924 | 0.907 |
|  |  | Morans’I | 0.563^***^ | 0.570^***^ | -0.015 |

Note: Moran’s I values are marked for statistical significance as follows: *** p < 0.001, ** 0.001 ≤ p < 0.01, * 0.01 ≤ p < 0.05, and no mark indicates p ≥ 0.05.

**Fig. S3.** Distribution of *β* and *R*² Estimates by Different Modelling Methods Across Multiple Scales

**Note S8.** Rationale for the window size and minimum-observation threshold.

Windowed regression, or local raster regression, estimates local model parameters within moving spatial neighborhoods rather than fitting a single global regression across the full study area. The choice of window size involves a trade-off between statistical stability and spatial specificity. Smaller windows can better preserve local variation but may contain too few valid observations and therefore produce unstable estimates. Larger windows provide more observations but may over-smooth spatial heterogeneity and obscure local variation in scaling relationships.

We selected the 30 km-by-30 km window size by balancing three considerations: ensuring a sufficient number of raster cells for local regression, avoiding excessive noise from overly small windows, and limiting over-smoothing from overly large windows. We also aimed to use a window size capable of capturing urban, suburban, and rural textures within local contexts, so that local regressions could reflect urban–suburban–rural gradients rather than only within-urban variation.

To further support this choice, we compared the window size with the spatial extent of Morphological Urban Areas (MUAs), which define contiguous settlement areas distinguishable from low-density peripheral and rural hinterlands. In the United States, the 75th percentile of MUA area is 803.7 km². Larger MUAs may also result from the physical overlap of two or more neighboring cities, which are merged and counted as a single urban agglomeration. This indicates that approximately 75% of contiguous urban settlements are smaller than 900 km², a spatial extent comparable to a 30 km-by-30 km window. Because this window size is comparable to or larger than the spatial extent of most MUAs, it is well suited to capturing combinations of urban, suburban, and rural areas in many local contexts. Although any moving-window approach inevitably partitions larger urban entities into local sections, a 30 km-by-30 km window provides a reasonable balance between statistical stability and spatial specificity.

At a 1 km-by-1 km raster resolution, each window contains up to 900 cells, providing sufficient potential observations for local regression while remaining fine enough to preserve subregional variation. We require a minimum of 10 valid observations for estimation. This threshold was used as a minimum inclusion criterion rather than as the typical sample size for local regressions. Its purpose was to exclude windows with extremely sparse valid data while retaining empirical variation in low-density areas.

We further tested the sensitivity of the results to the minimum number of valid cells required for model estimation. The lower threshold was set to 10 valid cells, and additional tests showed that increasing this threshold had little effect on the estimated results. As shown in Fig. S4, both *R*² and *β* tend to stabilize once the minimum number of valid cells exceeds 10.

We also recognize that this minimum-cell filtering involves a trade-off. Excluding windows with too few valid observations improves the stability of local regression estimates, but it also removes empirical observations, particularly in 30 km-by-30 km windows located in sparsely populated areas with limited population and infrastructure. Therefore, the threshold was selected to avoid highly unstable estimates while retaining as much empirical variation as possible across both low-density and high-density contexts.

**Fig. S4.** Sensitivity of estimated scaling exponents and model fit to the minimum-observation threshold in windowed regression.

**Note S9.** Rationale for population-density filter thresholds.

The purpose of applying population-density filters is to reduce the influence of uncertainties and potential outliers arising from both the original population data and the spatial transformation process. The use of multiple thresholds allows us to evaluate whether the estimated scaling exponents are sensitive to different filtering choices.

There are two main sources of uncertainty in the population gridded data. First, the original population data are derived from U.S. Census data at the census-block level. Although census data provide detailed population information, they may still contain uncertainty due to imputation procedures for missing information and data-protection methods that introduce statistical noise. Second, when the population vector data are transformed into regular grid cells, this transformation process may also introduce uncertainty, especially for grid cells with very small estimated populations.

As shown in Fig. S11 in Supplementary Note S12, the rank-frequency distribution of population density is approximately linear on a log-log scale, indicating that population density follows a strong power-law-like distribution. This means that the population gridded data contain a large number of cells with extremely low population values. These very low values may disproportionately affect the linear regression used in the scaling analysis, particularly if some of them are caused by statistical artifacts or spatial allocation uncertainty rather than by reliable observations.

Therefore, we applied population-density filters to reduce the potential influence of these extremely low-value cells while preserving as much of the population axis as possible. For a 1 km-by-1 km grid cell, a population value below and equal to 1 is difficult to interpret as a reliable population observation and is more likely to result from statistical artifacts or the vector-to-grid transformation process. We therefore used $\rho\leq1$ person/km^2^ as the lowest filtering threshold. In addition, based on our understanding of the data and the spatial resolution of the analysis, we considered $\rho\leq10$ people/km^2^ to be a reasonable upper threshold for identifying potentially unreliable low-population grid cells. We did not test thresholds higher than 10 because doing so would likely remove grid cells that represent valid low-density areas. Accordingly, we selected three thresholds, $\rho\leq1$ person/km^2^, $\rho\leq5$ people/km^2^, and $\rho\leq10$ people/km^2^, to examine the variability of the scaling results across different levels of filtering.

**Note S10.** Raw regression results of 18 variables.

Fig. S5 and Fig. S6 display the results with a population filter of 1 person/km² (P ≤ 1), Fig. S7 and Fig. S8 show the results with a population filter of 5 people/km² (P ≤ 5), and Fig. S9 and Fig. S10 illustrate the results with a population filter of 10 people/km² (P ≤ 10). The provided raw results include the distribution of population and response variables, as well as the regression coefficients (*β* values) and *R*^2^ values for the relationship between population density and density of response variables.

**Fig. S5.** Raw results of double log-linear regression with population filter of P ≤ 1.

The response variables analysed are total building mass, total building floor area, total building footprint, residential building mass, residential building floor area, residential building footprint, non-residential building mass, non-residential building floor area, and non-residential building footprint.

**Fig. S6.** Raw results of double log-linear regression with population filter of P ≤ 1.

The response variables analysed are total mass, total mobility infrastructure mass, total mobility infrastructure area, street mass, street area, on-road emissions, residential buildings emissions, commercial & industrial buildings emissions, and commercial buildings emissions.

**Fig. S7.** Raw results of double log-linear regression with population filter of P ≤ 5.

The response variables analysed are total building mass, total building floor area, total building footprint, residential building mass, residential building floor area, residential building footprint, non-residential building mass, non-residential building floor area, and non-residential building footprint.

**Fig. S8.** Raw results of double log-linear regression with population filter of P ≤ 5.

The response variables analysed are total mass, total mobility infrastructure mass, total mobility infrastructure area, street mass, street area, on-road emissions, residential buildings emissions, commercial & industrial buildings emissions, and commercial buildings emissions.

**Fig. S9.** Raw results of double log-linear regression with population filter of P ≤ 10.

The response variables analysed are total building mass, total building floor area, total building footprint, residential building mass, residential building floor area, residential building footprint, non-residential building mass, non-residential building floor area, and non-residential building footprint.

**Fig. S10.** Raw results of double log-linear regression with population filter of P ≤ 10.

The response variables analysed are total mass, total mobility infrastructure mass, total mobility infrastructure area, street mass, street area, on-road emissions, residential buildings emissions, commercial & industrial buildings emissions, and commercial buildings emissions.

**Note S11.** Calculate the error bar (confidence interval for scaling exponents).

We use the following equation to calculate the 95% confidence interval (CI) for scaling exponent $\beta$, which represents the uncertainty in the estimated scaling exponents:

$CI=\beta\pm t_{\alpha/2,df}\cdot{SE}_{\beta}$,

(4)

where ${SE}_{\beta}$ is the standard error of the parameter *β*, $t_{\alpha/2,df}$ represents the critical value from the t-distribution based on the significance level *α* (here, 0.05) and degrees of freedom (*df* = *n*−2), and *n* is the number of data points.

Table S10 presents the confidence interval (CI) and effective sample size for cases with population density filters of ≤1 person/km² and ≤10 people/km².

**Table S10.** Calculation results for confidence interval for scaling exponents.

| **Variables** | Population density filter ≤ 1 person/km^2^ | | | Population density filter ≤ 10 people/km^2^ | | |
| --- | --- | --- | --- | --- | --- | --- |
|  | LB | UB | Sample size | LB | UB | Sample size |
| Total mass | 0.62504 | 0.62650 | 3322320 | 0.78611 | 0.78828 | 1589868 |
| Total building mass | 0.83838 | 0.84004 | 2799837 | 0.95577 | 0.95812 | 1536370 |
| Residential building mass | 0.81203 | 0.81361 | 2783833 | 0.90328 | 0.90554 | 1532168 |
| Non-residential building mass | 0.60303 | 0.60640 | 1293469 | 0.82843 | 0.83375 | 838286 |
| Total mobility infrastructure mass | 0.45701 | 0.45848 | 3253667 | 0.61315 | 0.61544 | 1574037 |
| Street mass | 0.39000 | 0.39139 | 3237369 | 0.49904 | 0.50118 | 1570682 |
| Total building footprint | 0.77582 | 0.77728 | 2799636 | 0.89215 | 0.89423 | 1536310 |
| Residential building footprint | 0.75807 | 0.75946 | 2783547 | 0.85411 | 0.85612 | 1532084 |
| Non-residential building footprint | 0.53064 | 0.53375 | 1291390 | 0.73627 | 0.74120 | 836947 |
| Total building floor area | 0.84384 | 0.84545 | 2753606 | 0.97353 | 0.97582 | 1530104 |
| Residential building floor area | 0.82233 | 0.82385 | 2739838 | 0.92898 | 0.93116 | 1526057 |
| Non-residential building floor area | 0.60613 | 0.60956 | 1245925 | 0.83951 | 0.84487 | 819208 |
| Total mobility infrastructure area | 0.40641 | 0.40764 | 3268834 | 0.56932 | 0.57119 | 1576616 |
| Street area | 0.35043 | 0.35160 | 3246877 | 0.47640 | 0.47814 | 1572397 |
| Residential buildings emissions | 0.59891 | 0.60018 | 2881567 | 0.76472 | 0.76702 | 1524574 |
| Commercial buildings emissions | 0.55473 | 0.55675 | 1464080 | 0.75781 | 0.76072 | 1063771 |
| Commercial and industrial buildings emissions | 0.54234 | 0.54432 | 1982353 | 0.76780 | 0.77089 | 1262997 |
| On-road emissions | 0.67461 | 0.67671 | 3126779 | 0.80866 | 0.81238 | 1536773 |

Note: LB is the lower bound of the confidence interval, and UB represents the upper bound.

**Table S11.** Comparison of grid-based scaling law results in urban and non-urban areas with different boundary definitions.

| **Variables** | **The GHS-UCDB boundary** | | | | **The USCB-UADS boundary** | | | | | |
| --- | --- | --- | --- | --- | --- | --- | --- | --- | --- | --- |
|  | **Urban** | | **Non-urban** | | **Urban** | | | **Non-urban** | | |
|  | ***β*** | ***R*^2^** | ***β*** | ***R*^2^** | ***β*** | | ***R*^2^** | ***β*** | | ***R*^2^** |
| Total mass | 0.257 | 0.183 | 0.694 | 0.423 | 0.453 | 0.297 | | 0.643 | 0.275 | |
| Total building mass | 0.376 | 0.267 | 0.900 | 0.529 | 0.589 | 0.368 | | 0.866 | 0.382 | |
| Residential building mass | 0.732 | 0.530 | 0.873 | 0.536 | 0.786 | 0.543 | | 0.845 | 0.386 | |
| Non-residential building mass | 0.059 | 0.001 | 0.633 | 0.194 | 0.302 | 0.027 | | 0.494 | 0.087 | |
| Total mobility infrastructure mass | 0.136 | 0.042 | 0.511 | 0.277 | 0.312 | 0.137 | | 0.457 | 0.155 | |
| Street mass | 0.231 | 0.131 | 0.434 | 0.235 | 0.318 | 0.195 | | 0.404 | 0.134 | |
| Total building footprint | 0.421 | 0.386 | 0.836 | 0.552 | 0.602 | 0.451 | | 0.805 | 0.403 | |
| Residential building footprint | 0.751 | 0.576 | 0.816 | 0.560 | 0.784 | 0.598 | | 0.792 | 0.410 | |
| Non-residential building footprint | 0.045 | 0.001 | 0.557 | 0.177 | 0.273 | 0.025 | | 0.430 | 0.077 | |
| Total building floor area | 0.415 | 0.326 | 0.909 | 0.552 | 0.619 | 0.405 | | 0.869 | 0.401 | |
| Residential building floor area | 0.739 | 0.572 | 0.885 | 0.557 | 0.804 | 0.570 | | 0.855 | 0.409 | |
| Non-residential building floor area | 0.037 | 0.001 | 0.636 | 0.196 | 0.307 | 0.028 | | 0.495 | 0.088 | |
| Total mobility infrastructure area | 0.166 | 0.084 | 0.465 | 0.318 | 0.323 | 0.203 | | 0.411 | 0.179 | |
| Street area | 0.257 | 0.272 | 0.404 | 0.282 | 0.348 | 0.354 | | 0.369 | 0.156 | |
| Residential buildings emissions | 0.614 | 0.228 | 0.687 | 0.383 | 0.695 | 0.252 | | 0.596 | 0.240 | |
| Commercial buildings emissions | 0.282 | 0.062 | 0.877 | 0.381 | 0.584 | 0.182 | | 0.757 | 0.226 | |
| Commercial and industrial buildings emissions | 0.147 | 0.016 | 0.850 | 0.323 | 0.460 | 0.107 | | 0.743 | 0.188 | |
| On-road emissions | 0.146 | 0.010 | 0.697 | 0.247 | 0.427 | 0.071 | | 0.577 | 0.128 | |

**Note S12.** Statistics distribution of population density at different grid cell scales.

To understand the population statistics distribution in grid cell level, we examine the relationship between population density and its frequency Fig. S11), as well as the relationship between population density rank and the population density value Fig. S12), respectively. Both were plotted in the double-log format. These two analyses will provide a comprehensive overview of the statistical distribution of population density based on grid cells.

**Fig. S11.** Double-Logarithmic Plot of Population Density vs. Frequency.

Double-log plot of population density vs. frequency of appearance. We tested grid divisions ranging from 1 km to 9 km. All grids with a population density greater than 1 person/km² were sorted from low to high and divided into 60 equally spaced bins. The average population density of each bin is represented on the x-axis, and the number of grid cells within each density interval is represented on the y-axis.

**Fig. S12.** Double-Logarithmic Plot of Population Density Rank vs. Population Density.

Grid divisions were tested at scales ranging from 1 km to 9 km. All cells with a population density greater than 1 person/km² were sorted in descending order. The rank of each grid cell forms the x-axis, while the corresponding population density forms the y-axis.

**Self-similarity.** Fig. S11 and Fig. S12 show that the regression coefficients are similar across different grid cell sizes. This indicates that settlement units, when divided into grids of varying sizes, exhibit a consistent density distribution pattern, reflecting the self-similarity of human settlement distributions. This self-similarity likely explains the observed consistency in scaling exponents (*β* values) across different scales.

**Pareto distribution.** Fig. S11 illustrates a Pareto distribution of population density versus its frequency, reflecting the “few vital and many trivial” phenomenon. This distribution shows that as population density increases, its frequency decreases sharply. Consequently, grids with high population densities are relatively rare, while most areas have lower population densities. This pattern underscores the significant disparity in population density between urban and rural areas. Previous research suggests that a Pareto distribution of population density may be a prerequisite for the manifestation of urban scaling laws (Arcaute & Hatna, 2020). Therefore, our findings provide strong support for the validity and applicability of density scaling laws as analysed through grid-based methods.

In Fig. S12, we observe that when grid cells are ranked by population density from highest to lowest, the density decreases gradually among the top 100,000 grid cells. This gradual decline suggests that there is mild variation in population density between adjacent ranks within this subset. Conversely, the remaining 900,000+ grid cells exhibit a more pronounced and rapid decline in density. This contrast highlights that population density heterogeneity is considerably less in high-density areas compared to lower-density areas. Such findings help elucidate why scaling laws are less evident in densely populated urban centres.

# Fig. S13. Spatial distribution of scaling exponents. The response variables analysed are associated with material mass and footprints in building and mobility infrastructures.

**Fig. S14.** Spatial distribution of scaling exponents. The response variables related to the service areas of buildings and mobility infrastructures, as well as GHG emissions from various sectors.

**Fig. S15.** Mean values of Windowed linear regression using different population density filter.

**Note S13.** Evaluation of regional differences using parametric and non-parametric tests.

In the windowed linear regression analysis, we compared the regression results across the four U.S. census regions defined by the U.S. Census Bureau: Northeast, Midwest, South, and West (*Census Regions and Divisions of the United States*, n.d.). To evaluate whether regional differences were statistically significant, we applied several statistical tests here. (1) Levene’s test was used to assess the homogeneity of variance across regions, which is a key assumption of traditional one-way ANOVA. We found that this assumption was not satisfied for the raster-derived variables, Welch’s ANOVA was used as a more robust alternative. Welch’s ANOVA adjusts the test statistic and degrees of freedom and does not assume equal variances across groups. When significant regional differences were detected, Games-Howell post-hoc tests were conducted to identify which pairs of regions differed. (2) As a complementary non-parametric approach, the Kruskal-Wallis test was also applied. Unlike ANOVA-based methods, Kruskal-Wallis compares the ranks of observations rather than raw mean values and does not require normality or homogeneity of variance. Pairwise non-parametric post-hoc tests were then used to examine regional pairwise differences.

Given the large sample size of raster datasets, statistical significance was interpreted together with effect sizes, including partial eta-squared for Welch’s ANOVA and epsilon-squared for Kruskal-Wallis, to assess the practical magnitude of regional differences.

**Results of Levene’s test**

Levene’s test is a statistical method used to assess the equality of variances (homogeneity of variance) for a variable calculated for two or more groups. It is a critical prerequisite for many parametric tests, such as the standard One-Way ANOVA, which assumes that the variance within each group is approximately equal.

As shown in the table below, the Levene’s test results for all indicators significantly rejected the null hypothesis of homogeneity of variances (p < 0.001). As a result, this study employed Welch’s ANOVA and the non-parametric Kruskal-Wallis test, both of which are robust to heteroscedasticity (unequal variances), as alternatives to the standard One-Way ANOVA.

**Table S12.** Levene’s test for homogeneity of variances.

| Indicator | Levene Stat | P-Value | Is Homogeneous |
| --- | --- | --- | --- |
| Total mass | 128.737 | *** | No |
| Total building mass | 157.991 | *** | No |
| Residential building mass | 225.938 | *** | No |
| Non-residential building mass | 74.539 | *** | No |
| Total mobility infrastructure mass | 120.533 | *** | No |
| Street mass | 111.167 | *** | No |
| Total building footprint | 148.864 | *** | No |
| Residential building footprint | 219.423 | *** | No |
| Non-residential building footprint | 76.527 | *** | No |
| Total building floor area | 189.554 | *** | No |
| Residential building floor area | 255.511 | *** | No |
| Non-residential building floor area | 74.946 | *** | No |
| Total mobility infrastructure area | 84.205 | *** | No |
| Street area | 35.478 | *** | No |
| Residential buildings emissions | 88.683 | *** | No |
| Commercial buildings emissions | 18.608 | *** | No |
| Commercial & industrial emissions | 27.496 | *** | No |
| On-road emissions | 109.734 | *** | No |

Note: *** refers to p < 0.001.

**Results of Welch’s ANOVA**

Welch’s ANOVA was selected because Levene’s test indicated that the assumption of homogeneity of variance was violated across the four census regions. Compared with traditional one-way ANOVA, Welch’s ANOVA is more appropriate under unequal variances because it adjusts the test statistic and degrees of freedom.

The results show that all indicators exhibit statistically significant regional differences at the p < 0.001 level. However, the effect sizes (η^2^) are generally small, indicating that although regional differences are statistically detectable, their practical magnitude varies across indicators. The largest effect sizes are observed for residential building floor area (η² = 0.051), total building floor area (η² = 0.0393), residential building footprint (η² = 0.038), and residential building mass (η² = 0.0367). These results suggest that regional variation is more pronounced for residential building-related indicators than for non-residential building indicators or building emissions. In contrast, indicators such as non-residential building floor area (η² = 0.0021), residential buildings emissions (η² = 0.003), and non-residential building mass (η² = 0.0055) show very small effect sizes, suggesting limited practical regional differentiation despite statistical significance.

Regarding mobility infrastructure and street-related metrics, the results also indicate statistically significant regional differences, but their effect sizes are generally small to moderate. Among these indicators, total mobility infrastructure area shows the largest regional effect (η² = 0.0343), followed by on-road emissions (η² = 0.0318), total mobility infrastructure mass (η² = 0.0217), and street mass (η² = 0.0204). In contrast, street area shows a smaller effect size (η² = 0.0131). This suggests that regional differences in mobility-related infrastructure are more evident in total infrastructure area and mass, as well as on-road emissions, than in street area alone.

**Table S13.** Welch’s ANOVA Test Results for Windowed Regression Indicators Across Census Regions.

| Indicator | F statistic | P-Value | Eta Squared (η^2^) |
| --- | --- | --- | --- |
| Total mass | 114.6 | *** | 0.033 |
| Total building mass | 136.432 | *** | 0.029 |
| Residential building mass | 194.854 | *** | 0.0367 |
| Non-residential building mass | 12.248 | *** | 0.0055 |
| Total mobility infrastructure mass | 86.525 | *** | 0.0217 |
| Street mass | 93.923 | *** | 0.0204 |
| Total building footprint | 137.808 | *** | 0.0302 |
| Residential building footprint | 195.841 | *** | 0.038 |
| Non-residential building footprint | 17.177 | *** | 0.0077 |
| Total building floor area | 159.421 | *** | 0.0393 |
| Residential building floor area | 231.601 | *** | 0.051 |
| Non-residential building floor area | 6.24 | *** | 0.0021 |
| Total mobility infrastructure area | 104.582 | *** | 0.0343 |
| Street area | 34.634 | *** | 0.0131 |
| Residential buildings emissions | 6.924 | *** | 0.003 |
| Commercial buildings emissions | 20.781 | *** | 0.0109 |
| Commercial & industrial emissions | 29.434 | *** | 0.0142 |
| On-road emissions | 97.066 | *** | 0.0318 |

Note: *** refers to p < 0.001. F statistic measures how large the differences among group means are relative to the variation within groups, i.e. whether the mean scaling exponents differ across regions. A larger F statistic generally indicates stronger evidence that at least one regional mean differs from the others. Eta squared is an effect size measure. It indicates how much of the variation in the dependent variable is associated with regional grouping. E.g., η² = 0.02 means that about 2% of the variation in scaling exponents is associated with regional differences.

Games-Howell post-hoc tests were conducted for the indicators with the largest effect sizes in Welch’s ANOVA to identify which pairs of census regions differed significantly. The results show that most regional pairs exhibit statistically significant differences, although the direction and magnitude of these differences vary by indicator.

For residential building mass and residential building footprint, all pairwise regional comparisons are significant. The Midwest has lower mean values than the Northeast, South, and West, as indicated by the negative mean differences. The Northeast and South both show higher values than the West, while the Northeast is also slightly higher than the South. This suggests a clear regional gradient in residential building-related mass and footprint indicators.

For total building floor area and residential building floor area, most regional pairs are also significantly different. However, the comparison between the Midwest and West is not significant for either indicator, with p = 0.997 in both cases. This indicates that Midwest and West have statistically similar values for these floor-area indicators, while both regions differ significantly from the Northeast and South.

For total mobility infrastructure area, all pairwise comparisons are statistically significant. The Midwest is lower than the Northeast and South, but higher than the West. The Northeast shows the largest positive differences relative to the other regions, especially compared with the West. This suggests that regional differentiation in mobility infrastructure area is also pronounced, with the Northeast showing consistently higher values and the West lower values.

Overall, the Games-Howell results indicate that the significant effects detected by Welch’s ANOVA are not driven by a single regional contrast. Instead, regional differences are widespread across most pairwise comparisons, particularly for residential building indicators and total mobility infrastructure area. However, the non-significant Midwest–West comparisons for building floor area suggest that some regional similarities remain, even among indicators with relatively large overall effect sizes.

**Table S14.** Games-Howell Post-hoc Pairwise Comparisons of Regional Differences for Selected Indicators.

| Indicator | Group A | Group B | Mean Diff | T-Stat | P-Value |
| --- | --- | --- | --- | --- | --- |
| Residential building mass | Midwest | Northeast | -0.0948 | -18.95 | *** |
|  | Midwest | South | -0.08 | -20.76 | *** |
|  | Midwest | West | -0.0266 | -4.44 | *** |
|  | Northeast | South | 0.0148 | 2.99 | ** |
|  | Northeast | West | 0.0682 | 10.13 | *** |
|  | South | West | 0.0534 | 9.01 | *** |
| Residential building footprint | Midwest | Northeast | -0.0891 | -19.1 | *** |
|  | Midwest | South | -0.0751 | -20.66 | *** |
|  | Midwest | West | -0.0235 | -4.26 | *** |
|  | Northeast | South | 0.014 | 3.03 | ** |
|  | Northeast | West | 0.0655 | 10.55 | *** |
|  | South | West | 0.0516 | 9.41 | *** |
| Total building floor area | Midwest | Northeast | -0.0863 | -15.6 | *** |
|  | Midwest | South | -0.0692 | -17.74 | *** |
|  | Midwest | West | 0.0012 | 0.2 | 0.997 |
|  | Northeast | South | 0.017 | 3.2 | ** |
|  | Northeast | West | 0.0874 | 12.71 | *** |
|  | South | West | 0.0704 | 12.45 | *** |
| Residential building floor area | Midwest | Northeast | -0.101 | -19.31 | *** |
|  | Midwest | South | -0.0849 | -21.93 | *** |
|  | Midwest | West | -0.0013 | -0.2 | 0.997 |
|  | Northeast | South | 0.0161 | 3.25 | ** |
|  | Northeast | West | 0.0998 | 14.25 | *** |
|  | South | West | 0.0837 | 13.82 | *** |
| Total mobility infrastructure area | Midwest | Northeast | -0.0666 | -12.05 | *** |
|  | Midwest | South | -0.0156 | -4.14 | *** |
|  | Midwest | West | 0.0391 | 8.04 | *** |
|  | Northeast | South | 0.051 | 9.59 | *** |
|  | Northeast | West | 0.1058 | 17.17 | *** |
|  | South | West | 0.0547 | 11.8 | *** |

Note: *** indicates p < 0.001; ** indicates p < 0.05. Mean Difference is the difference between the mean values of two groups being compared. If Mean Diff > 0, the first region has a higher mean scaling exponent than the second region. T statistic measures how large the mean difference is relative to the uncertainty or variability of that pairwise comparison. A larger absolute T-Stat means the difference between the two regional means is large relative to the variability in the data.

**Results of Kruskal-Wallis Tests**

To further confirm the differences. As an alternative, we also ran Kruskal-Wallis tests. The results show that all indicators exhibit statistically significant regional differences at the p < 0.001 level. However, as with Welch’s ANOVA, the strength of these differences varies substantially across indicators. The largest effect sizes are observed for residential building floor area (ε² = 0.1047), residential building mass (ε² = 0.1039), residential building footprint (ε² = 0.1012), and total building mass (ε² = 0.0836). This confirms that residential building-related indicators show the strongest regional differentiation.

Building-related indicators generally have larger effect sizes than emissions-related indicators. For example, total building footprint (ε² = 0.0795) and total building floor area (ε² = 0.0787) show relatively pronounced regional differences, whereas residential buildings emissions (ε² = 0.0032), commercial buildings emissions (ε² = 0.0114), and commercial & industrial emissions (ε² = 0.0154) show much weaker regional effects.

Mobility infrastructure and street-related indicators also show significant but more moderate regional differentiation. Among these, total mobility infrastructure area (ε² = 0.0410), street mass (ε² = 0.0352), street area (ε² = 0.0345), and on-road emissions (ε² = 0.0325) have larger effect sizes than total mobility infrastructure mass (ε² = 0.0284), but remain below the leading residential building indicators.

Overall, the Kruskal-Wallis results are consistent with the Welch’s ANOVA results: regional differences are statistically significant across all indicators, but their practical magnitude is strongest for residential building-related variables and weaker for emissions-related variables.

**Table S15.** Kruskal-Wallis Test Results for Regional Differences Across Indicators.

| Indicator | H-Stat | P-Value | Epsilon Squared (ϵ^2^) |
| --- | --- | --- | --- |
| Total mass | 377.21 | *** | 0.0461 |
| Total building mass | 677.448 | *** | 0.0836 |
| Residential building mass | 835.686 | *** | 0.1039 |
| Non-residential building mass | 95.643 | *** | 0.0126 |
| Total mobility infrastructure mass | 231.986 | *** | 0.0284 |
| Street mass | 287.503 | *** | 0.0352 |
| Total building footprint | 644.348 | *** | 0.0795 |
| Residential building footprint | 813.958 | *** | 0.1012 |
| Non-residential building footprint | 114.318 | *** | 0.0151 |
| Total building floor area | 632.504 | *** | 0.0787 |
| Residential building floor area | 835.949 | *** | 0.1047 |
| Non-residential building floor area | 26.698 | *** | 0.0036 |
| Total mobility infrastructure area | 334.817 | *** | 0.041 |
| Street area | 281.136 | *** | 0.0345 |
| Residential buildings emissions | 19.196 | *** | 0.0032 |
| Commercial buildings emissions | 61.998 | *** | 0.0114 |
| Commercial & industrial emissions | 88.79 | *** | 0.0154 |
| On-road emissions | 262.887 | *** | 0.0325 |

Note: *** refers to p < 0.001. H-Stat measures whether the rank distributions differ across groups. A larger H statistic generally indicates stronger evidence that at least one region differs from the others. Epsilon squared is an effect size measure. It indicates how much variation in the ranked scaling exponents can be explained by census region.

Pairwise non-parametric post-hoc tests were conducted for the indicators with the largest Kruskal-Wallis effect sizes to identify which regional pairs differed significantly. The results show that most pairwise regional comparisons are statistically significant, but several Northeast–South comparisons are not significant after adjustment.

For total building mass, residential building mass, total building footprint, and residential building footprint, the Midwest is significantly lower than the Northeast, South, and West. The Northeast and South are not significantly different from each other for these indicators, suggesting similar distributions between these two regions. However, both the Northeast and South are significantly higher than the West.

For residential building floor area, most pairwise comparisons are also significant. The Midwest is significantly lower than the Northeast and South, while the difference between the Midwest and West is statistically significant but has an extremely small effect size (Hedges’ g = -0.006), indicating that this difference is practically negligible. The Northeast–South comparison is not significant, whereas both regions differ significantly from the West.

Overall, the pairwise non-parametric results are consistent with the Kruskal-Wallis tests and broadly align with the Welch’s ANOVA and Games-Howell results. Regional differences are most evident between the Midwest and the Northeast/South, and between the West and the Northeast/South. In contrast, the Northeast and South often show similar values for building-related indicators.

**Table S16.** Pairwise non-parametric post-hoc comparisons for indicators with the largest Kruskal-Wallis effect sizes

| Indicator | Group A | Group B | P-Value (adj) | Hedges’g |
| --- | --- | --- | --- | --- |
| Total building mass | Midwest | Northeast | *** | -0.615 |
|  | Midwest | South | *** | -0.505 |
|  | Midwest | West | *** | -0.153 |
|  | Northeast | South | 1 | 0.078 |
|  | Northeast | West | *** | 0.229 |
|  | South | West | *** | 0.206 |
| Residential building mass | Midwest | Northeast | *** | -0.736 |
|  | Midwest | South | *** | -0.584 |
|  | Midwest | West | *** | -0.127 |
|  | Northeast | South | 0.653 | 0.112 |
|  | Northeast | West | *** | 0.284 |
|  | South | West | *** | 0.259 |
| Total building footprint | Midwest | Northeast | *** | -0.636 |
|  | Midwest | South | *** | -0.498 |
|  | Midwest | West | *** | -0.153 |
|  | Northeast | South | 0.093 | 0.119 |
|  | Northeast | West | *** | 0.258 |
|  | South | West | *** | 0.209 |
| Residential building footprint | Midwest | Northeast | *** | -0.735 |
|  | Midwest | South | *** | -0.581 |
|  | Midwest | West | *** | -0.122 |
|  | Northeast | South | 0.288 | 0.112 |
|  | Northeast | West | *** | 0.297 |
|  | South | West | *** | 0.27 |
| Residential building floor area | Midwest | Northeast | *** | -0.733 |
|  | Midwest | South | *** | -0.624 |
|  | Midwest | West | *** | -0.006 |
|  | Northeast | South | 0.14 | 0.13 |
|  | Northeast | West | *** | 0.403 |
|  | South | West | *** | 0.403 |

Note: *** refers to p < 0.001. Hedges’ g is an effect size measure to assess the practical magnitude of pairwise regional differences. Hedges’ g represents the standardized difference between two groups, corrected for small-sample bias. Positive values indicate that the first region has a higher average scaling exponent than the second region, whereas negative values indicate the opposite. Larger absolute values of Hedges’ g indicate larger practical differences between regions.

**Note S14.** Grid-based scaling laws across different grid cell sizes.

Here we look for systematic variations of *β* with the definitional grid cell size, and ways to interpret and explain them.

**Fig. S16.** The comprehensive results of the grid-based scaling law tested across 20 different grid cell sizes. **a** illustrates the scaling efficiency for 18 different variables, whereas **b** presents the corresponding *R*² values. The horizontal axis represents the variation in grid size from 1 km to 20 km in 1 km intervals.

As depicted in Fig. S16, firstly, as the grid cell size increases, the scaling relationship remains present, and the goodness of fit (*R*² value) of the scaling relationship also improves. This indicates that with increasing granularity, the explanatory power of the power-law model is enhanced. Specifically, mobility infrastructure-related variables demonstrate a slightly decreasing trend in *β* values as grid size increases, eventually stabilizing. In contrast, building-related variables exhibit an opposite trend, with *β* values increasing until saturation as granularity increases.

The scaling relationship between various metrics and population exhibits only minor variation across different grid cell sizes, indicating that the scaling exponents (*β*) remain consistently stable across various grid cell sizes. The observed minor fluctuations in scaling exponents are likely due to changes in the granularity of observation grid sizes, which can affect the statistical distribution of the response variables. Specifically, smaller grid scales may include numerous outliers with low-density values, whereas larger grid scales could compress the overall range of these values. For mobility infrastructure-related variables, using larger observation units compresses the range of density variation, leading to smaller *β* values. Conversely, for building-related variables, although there is also a compression of the value range, many smaller observation units affect the statistical distribution of the data, which is reflected in initially smaller *β* values.

As shown in Table S17, when using a 1 km-by-1 km grid cell size, the total mobility infrastructure mass varies by a factor of 23-fold from the 25th to the 95th percentile. In contrast, with a 12 km-by-12 km grid cell size, it varies by a factor of 11-fold. Conversely, for total building mass, the variation from the 25th to the 95th percentile is 87-fold for the 1 km-by-1 km grid and 273-fold for the 12 km-by-12 km grid.

**Table S17.** Statistical description of total mobility infrastructure mass and total building mass for 1km-by-1km and 12 km-by-12 km grid cell sizes.

| **Response variables** | **Grid cell size** | **25% quantile** | **50% quantile** | **75% quantile** | **95% quantile** |
| --- | --- | --- | --- | --- | --- |
| **Total mobility infrastructure mass (t/km^2^)** | 1km-by-1km | 3244.5 | 6078.7 | 12889.4 | 74942.5 |
|  | 12km-by-12km | 2345.6 | 4651.6 | 8489.8 | 28013.8 |
| **Total building mass (t/km^2^)** | 1km-by-1km | 877.7 | 2541.6 | 7455.9 | 76979.2 |
|  | 12km-by-12km | 87.7 | 753.0 | 3307.6 | 23992.3 |

The primary reason for the lower *R*² values observed when using smaller grid cells as the observation unit can be largely explained by the spatial decoupling effect between physical structures and residential populations. This decoupling effect becomes more prominent at finer spatial resolutions, as smaller grid units increase the possibility of mismatches between infrastructure placement and recorded population data. The population data used in this study is derived from the U.S. Census Bureau’s census blocks, which represent the “nighttime population,” meaning individuals are recorded at their residential location rather than daytime activity patterns. In extreme cases, a 1 km² space may contain very few residents, but a substantial amount of infrastructure, such as in industrial zones or transportation hubs. As grid size increases, data aggregation naturally smooths out the effects of spatial decoupling, reducing the high variability introduced by the decoupling effect and leading to a lower overall variability in the data. This typically results in a higher *R*² value. Importantly, while larger grids yield higher *R*² values, the consistency of scaling exponents across different grid sizes demonstrates that the fundamental relationships remain robust, indicating that lower *R*² values at finer spatial resolutions reflect the spatial decoupling effect rather than reduced model reliability.

**Note S15.** Comparisons of results using total amount vs. density values in a grid-based scaling model.

|  |  |
| --- | --- |
| (a) | (b) |

# Fig. S17. Cumulative distribution plots of urban areas defined by (a) MUA (Taubenböck et al., 2019) and (b) GHS-FUA (Schiavina et al., 2019).

**Table S18.** Summary statistics of urban area distributions under different urban definitions in the United States.

|  | MUA (km^2^) | GHS-FUA (km^2^) |
| --- | --- | --- |
| Mean | 729.76 | 2194.14 |
| Min | 57.10 | 93.00 |
| 25% | 299.24 | 678.00 |
| 50% | 445.72 | 1224.50 |
| 75% | 803.70 | 2464.75 |
| Max | 6148.53 | 19826.00 |

**Note S16.** Interpretation of fractal dimensions and their application to our case

Fractal dimension is a general measure of how a spatial pattern fills space across scales. In the context of this study, the fractal dimension of infrastructure refers to the extent to which infrastructure, such as building footprints, street networks, or street area, occupies 2D or 3D space across scales. A more compact and space-filling infrastructure pattern would correspond to a higher fractal dimension, whereas a more fragmented, sparse, or linear pattern would correspond to a lower fractal dimension.

If one were to estimate the fractal dimension of infrastructure from spatial data, a commonly used approach would be box-counting. Specifically, one could take the infrastructure geometry inside each 1 km-by-1 km cell, overlay smaller sub-boxes, for example 10 m, 25 m, 50 m, 100 m, 200 m, and 500 m, count the number of sub-boxes that intersect the infrastructure, $N(\epsilon)$, and estimate:

$$D=\frac{\Delta logN(\epsilon)}{\Delta log(1/\epsilon)},$$

(5)

where $\epsilon$ is the sub-box size, $N(\epsilon)$ is the number of occupied sub-boxes, and $D$ is the box-counting fractal dimension.

For population dimension, population size itself does not have a fractal dimension. Rather, it is the spatial distribution of population that can be analyzed using fractal concepts, if that distribution shows scale-dependent clustering, heterogeneity, or self-similar spatial structure. Following Molinero and Thurner (2021), the population fractal dimension can be divided into two components: the projected population dimension, $d_{pp}$, which describes the two-dimensional distribution of population on the ground surface, and the three-dimensional population dimension, $d_{p}$, which describes the estimated distribution of people within buildings. Because true three-dimensional population locations are usually unavailable, Molinero and Thurner approximate the vertical component using building-height information:

$$d_{p}=d_{pp}+\eta=d_{pp}+\frac{\log\langle h\rangle}{\log h_{m}},$$

(6)

where $\langle h\rangle$ is the average number of building levels and $h_{m}$ is the maximum number of building levels. Thus, in gridded data, $d_{pp}$ could in principle be estimated from the projected population distribution using a box-counting method, while estimating $d_{p}$ would require additional information on building height, floors, or the vertical distribution of residential or occupied floor space.

We note that although estimating local fractal dimensions at grid-cell level is theoretically applicable, our analysis does not directly calculate these local fractal dimensions. Instead, we use the fractal-dimension framework of Molinero and Thurner (2021) as a theoretical basis for interpreting the scaling exponents observed in our gridded scaling analysis. Combined with the finding by Carbone et al. (2022) that fractal dimension is scale-dependent in empirical data, that is, urban fractal dimensions vary with the scale of observation, this framework supports our interpretation that changes in grid-cell size may lead to variations in the observed scaling relationships.

**Note S17.** Theoretical Modelling of Grid-Level Sublinear Scaling Based on Fractal Dimensions.

Molinero’s (Molinero & Thurner, 2021) theoretical formulation states that for spatially distributed variables such as infrastructure and population, the scaling exponent $\beta$ can be expressed as the ratio of their fractal dimensions:

$$\beta=\frac{d_{i}}{d_{p}}.$$

(7)

According to Molinero’s (Molinero & Thurner, 2021), in a grid-based spatial framework, each grid cell of side length $L$ serves as a standardized measurement unit. The infrastructure variable $I(L)$ (e.g., road length or building footprint) within a grid cell can be expressed as:

$$I(L)\propto L^{d_{i}}.$$

(8)

Similarly, the population aggregated within the same cell scales as:

$$P(L)\propto L^{d_{p}}.$$

(9)

The measured scaling exponent $\beta$, therefore, emerges from the ratio of these aggregated quantities across grids:

$$I(L)\propto[P\left( L \right)]^{\beta}.$$

(10)

Substituting the above equations gives the relationship:

$$L^{d_{i}}\propto\left( L^{d_{p}} \right)^{\beta}.$$

(11)

This directly implies:

$$L^{d_{i}}\propto L^{\beta d_{p}} \Rightarrow d_{i}=\beta d_{p}.$$

(12)

Solving for $\beta$:

$$\beta=\frac{d_{i}}{d_{p}}.$$

(13)

For population, Molinero notes that people occupy three-dimensional buildings, and therefore the fractal dimension of the population $d_{p}$ should be greater than that of the road network, i.e., $d_{p}>2$. If we conceptualize the “population points” (each person having a 3D coordinate) in a city as forming a fractal in space, its three-dimensional fractal dimension $d_{p}$ can be expressed as:

$$d_{p}=d_{p}^{(2D)}+d_{v},$$

(14)

where $d_{p}^{(2D)}$ is the projected (planar) fractal dimension of the population distribution, and $d_{v}$ represents the vertical contribution from building heights (number of floors).

The grid-level scaling exponent $\beta_{\text{sub}}$ then follows:

$$\beta_{\text{sub}}=\frac{d_{i}}{d_{p}}<1.$$

(13)

This inequality reflects the fact that $d_{i}<d_{p}$, producing sublinear scaling between infrastructure and population.

**Scale Dependence and Effects of Grid Aggregation**

When moving from city-level to grid-level measurements, fractal dimensions become scale dependent due to spatial aggregation effects: (1) Infrastructure ($d_{i}$). At small grid sizes (e.g., $1 \text{km}^{2}$), networks appear fragmented, resulting in a lower measured $d_{i}(L)$ than at the city scale. As grid size increases, $d_{i}(L)$ approaches its city-scale value. (2) Population ($d_{p}$). The measured $d_{p}(L)$ remains closer to its city-scale value, because vertical extensions of buildings contribute an additional dimension that is less sensitive to horizontal subdivision.

We can express these scale-dependent effects as:

$$d_{i}(L)\leq d_{i}^{\text{(city)}}, d_{p}(L)\approx d_{p}^{\text{(city)}}.$$

(15)

Therefore, the grid-based scaling exponent satisfies:

$$\beta_{\text{sub}}(L)=\frac{d_{i}(L)}{d_{p}(L)}<\frac{d_{i}^{\text{(city)}}}{d_{p}^{\text{(city)}}}=\beta_{\text{city}}.$$

(16)

This explains why grid-based measurements often yield slightly smaller $\beta$ values than city-based scaling, as sublinear relationships are accentuated due to the lower measured complexity of infrastructure in smaller, fragmented spatial units.

As $L$ increases, $d_{i}\left( L \right) converges to d_{i}^{\text{(city)}}$ and $\beta_{\text{sub}}\left( L \right) \mathrm{to}\beta_{\text{city}}$, providing a theoretical basis for the observed convergence of grid-based scaling toward city-based values at coarser resolutions.

**Note S18.** Stability of the combined scaling exponent for street area and building footprint.

We observed that the combined scaling exponents of street area and total building footprint remain approximately constant across grid sizes, ranging from 1.13 to 1.15 (Table S19). To evaluate whether this regularity reflects a stable relationship across spatial resolutions, we conducted a variance-propagation analysis. The primary objective was to assess whether the combined exponent, $\mathrm{SUM}_{\beta}=\beta_{\text{stree\_area}}+\beta_{\text{building\_footprint}}$, remains stable across varying grid sizes.

**Table S19.** Scaling exponents across grid sizes (population density ≤ 1 person/km²).

| **Grid size** | $\beta_{street\_area}$ | $\beta_{building\_footprint}$ | ${SUM}_{\beta}$ |
| --- | --- | --- | --- |
| 1km | 0.35 | 0.78 | 1.13 |
| 2km | 0.32 | 0.82 | 1.13 |
| 3km | 0.30 | 0.84 | 1.14 |
| 4km | 0.29 | 0.85 | 1.14 |
| 5km | 0.29 | 0.86 | 1.14 |
| 6km | 0.28 | 0.86 | 1.15 |
| 7km | 0.28 | 0.87 | 1.15 |
| 8km | 0.27 | 0.88 | 1.15 |
| 9km | 0.27 | 0.88 | 1.15 |
| 10km | 0.27 | 0.88 | 1.15 |
| 11km | 0.27 | 0.88 | 1.15 |
| 12km | 0.27 | 0.88 | 1.15 |
| 13km | 0.27 | 0.88 | 1.15 |
| 14km | 0.26 | 0.88 | 1.14 |
| 15km | 0.26 | 0.88 | 1.14 |
| 16km | 0.26 | 0.88 | 1.14 |
| 17km | 0.26 | 0.87 | 1.13 |
| 18km | 0.26 | 0.87 | 1.13 |
| 19km | 0.26 | 0.87 | 1.13 |
| 20km | 0.26 | 0.87 | 1.13 |

According to variance-propagation method, the variance of the combined exponent is given by:

$\mathrm{Var}\left( \mathrm{SUM}_{\beta} \right)=\mathrm{Var}\left( \beta_{\text{street\_area}} \right)+\mathrm{Var}\left( \beta_{\text{building\_footprint}} \right)+2\text{ }\mathrm{Cov}\left( \beta_{\text{street\_area}},\beta_{\text{building\_footprint}} \right)$.

(17)

This formulation shows that the stability of $\mathrm{SUM}_{\beta}$is determined not only by the variability of each individual exponent, but also by their covariance structure. A negative covariance (Cov < 0) indicates that the two exponents tend to vary in opposite directions across grid sizes, producing a compensatory effect that stabilizes their sum. In contrast, a positive covariance would indicate co-variation in the same direction and would amplify fluctuations in the combined exponent.

The results show a strong negative association between $\beta_{\text{street\_area}}$ and $\beta_{\text{building\_footprint}}$ across grid sizes, with a Pearson correlation coefficient of $R=-0.965$ and $p<0.001$ (Table S20). The covariance is also negative. As a result, the variance of $\mathrm{SUM}_{\beta}$ is substantially lower than the variances of the two individual exponents, indicating that the combined scaling structure is more stable across grid sizes than either component alone.

**Table S20.** Statistical summary of variance decomposition and correlation analysis.

| **Statistic** | **Value** |
| --- | --- |
| Building footprint Variance | 0.000687 |
| Street area Variance | 0.000516 |
| Covariance | -0.000575 |
| Combined Sum Variance | 0.000054 |
| Correlation | -0.965072 |
| Correlation coefficient (R): | -0.965072 |
| P-value | 0.000000 |

We further visualize this relationship in a two-dimensional state space, where each point represents a grid size and an iso-sum reference line is used to evaluate deviations from constant-sum behavior. As shown in Fig. S18, $\mathrm{SUM}_{\beta}$ fluctuates around approximately 1.14 across grid sizes from 1 km to 20 km. This pattern is consistent with the variance-propagation results and supports the interpretation of a compensatory relationship between the two exponents across spatial resolutions.

**Fig. S18. Relationship between the scaling exponents of street area and total building footprint across different spatial aggregation levels.** Each point represents a specific grid size, and the connected trajectory shows how the joint scaling structure evolves as spatial resolution changes from fine to coarse. The dashed line represents the iso-sum constraint, defined as a constant total scaling exponent, $\mathrm{SUM}_{\beta}\approx1.141$. Points lying on this line indicate perfect compensation between the two exponents. The “×” marker denotes the mean state of the system, that is, the average values of $\beta_{\text{street\_area}}$ and $\beta_{\text{building\_footprint}}$ across all grid sizes.

**Note S19.** Estimating population density in urban, suburban, and rural areas.

The benchmark densities used in the per-capita comparison are not direct thresholds from an official U.S. urban–rural classification. This is because official U.S. urban–rural classifications do not provide a separate and operational definition of “suburban” areas that can be directly used to estimate a suburban population-density benchmark. Therefore, we used the GHS-SMOD dataset (Global Human Settlement Layer – Settlement Model; Schiavina et al., 2022) to classify settlement types and estimate representative population densities for urban, suburban, and rural areas.

The GHS-SMOD dataset classifies settlement types using population size, population density, and built-up area. It provides gridded settlement classes, including urban centre, dense urban cluster, semi-dense urban cluster, suburban or peri-urban grid cell, rural cluster grid cell, low-density rural grid cell, and very low-density rural grid cell. In our analysis, we classified urban areas as cells labelled “urban centre” or “dense urban cluster”; suburban areas as cells labelled “semi-dense urban cluster” or “suburban or peri-urban grid cell”; and rural areas as all remaining settlement types.

To estimate representative population densities, we first identified effective population grid cells, defined as cells with population density >1 person/km², within each of the three categories. We then calculated the total population and corresponding settlement land area for each category by summing the population values and counting the associated 1 km² grid cells. The resulting average population densities were approximately 1,898 people/km² for urban areas, 688 people/km² for suburban areas, and 29 people/km² for rural areas (Table S21).

**Table S21.** Estimated population density in urban, suburban, and rural areas.

| **Classification** | **Total population**  (people) | **Total land area**  (km^2^) | **Population density**  (people/ km^2^) |
| --- | --- | --- | --- |
| Urban area | 151,149,870 | 79,645 | 1,898 |
| Suburban area | 79,393,400 | 115,358 | 688 |
| Rural area | 98,121,730 | 3,401,747 | 29 |

Based on these estimates, we selected simplified benchmark densities of 1,800 people/km² for urban units, 600 people/km² for suburban units, and 50 people/km² for rural units. These values are rounded benchmark densities used for convenient and transparent per-capita comparison. The rural benchmark was set to 50 people/km² as a conservative baseline close to, but slightly above the estimated rural average of 29 people/km². The suburban and urban benchmarks approximate the estimated GHS-SMOD-based densities while preserving simple multiples of the rural baseline: 600 people/km² is 12 times the baseline, and 1,800 people/km² is 36 times the baseline.

We use the same approach as described above to identify the total values for the selected indicators in rural areas and in urban areas (excluding suburban areas), as follows:

**Table S22.** Estimated total values of indicators in low-density rural areas and high-density urban areas.

| **Indicators** | **Total values in rural areas** | **Total values in urban areas** (excluding suburban areas) | **The differences between cities and rural areas** |
| --- | --- | --- | --- |
| Total mass | 58 Gt | 34 Gt | 24 Gt |
| Total building footprint | 13,105 km^2^ | 10,829 km^2^ | 2,276 km^2^ |
| Total mobility infrastructure area | 68,139 km^2^ | 21,381 km^2^ | 46,758 km^2^ |
| On-road emissions | 682 Mt CO_2_e | 519 Mt CO_2_e | 163 Mt CO_2_e |

**Table S23.** Per capita values of key indicators with different population densities.

| **Variables** | **Low density rural units (**$\boldsymbol{x}$**)** | **Medium density suburban units (**$\boldsymbol{12}\boldsymbol{x}$**)** | **High density urban units (**$\boldsymbol{36}\boldsymbol{x}$**)** | **Scaling exponents** |
| --- | --- | --- | --- | --- |
| Total mass | 100.00% | 49.93% | 36.73% | 0.72 |
| Total building mass | 100.00% | 80.06% | 72.56% | 0.91 |
| Residential building mass | 100.00% | 72.32% | 62.67% | 0.87 |
| Non-residential building mass | 100.00% | 50.84% | 37.70% | 0.73 |
| Total mobility infrastructure mass | 100.00% | 32.49% | 19.77% | 0.55 |
| Street mass | 100.00% | 25.82% | 14.19% | 0.46 |
| Total building footprint | 100.00% | 68.32% | 57.73% | 0.85 |
| Residential building footprint | 100.00% | 63.55% | 52.01% | 0.82 |
| Non-residential building footprint | 100.00% | 41.41% | 28.04% | 0.65 |
| Total building floor area | 100.00% | 82.53% | 75.81% | 0.92 |
| Residential building floor area | 100.00% | 75.77% | 67.02% | 0.89 |
| Non-residential building floor area | 100.00% | 51.92% | 38.86% | 0.74 |
| Total mobility infrastructure area | 100.00% | 28.91% | 16.70% | 0.50 |
| Street area | 100.00% | 23.93% | 12.72% | 0.42 |
| Residential buildings emissions | 100.00% | 54.12% | 41.26% | 0.75 |
| Commercial buildings emissions | 100.00% | 87.17% | 82.04% | 0.94 |
| Commercial and industrial buildings emissions | 100.00% | 80.04% | 72.53% | 0.91 |
| On-road emissions | 100.00% | 53.66% | 40.75% | 0.75 |

Note: the population densities of spatial units are expressed as a multiple of that of a baseline unit with a population density of *x*. In this study, we define population density benchmarks as 50 people/km² for rural units in the contig. U.S. (baseline), 600 people/km² for suburban units (12 times the baseline), and 1800 people/km² for urban units (36 times the baseline). The per capita key indicator values of those units are presented as percentages relative to the per capita value of the baseline unit.

**Note S20.** Temporal stability of cross-sectional scaling laws and scaling exponents.

Existing studies suggest that cross-sectional urban scaling exponents, denoted by β, are generally stable over time. Although the baseline levels of urban indicators, captured by the scaling prefactor $Y_{0}$, may change substantially in response to technological, economic, demographic, or institutional shifts, the relative relationship between city size and urban indicators often remains persistent (Bettencourt, 2013; Burghardt et al., 2024).

It is important to note that “stability” does not imply that scaling exponents remain identical across all periods. Rather, it means that the scaling relationship tends to preserve the same qualitative regime, such as superlinear, linear, or sublinear scaling, while the estimated exponent fluctuates within a relatively narrow range. In this sense, temporal stability refers to the persistence of the scaling pattern rather than the invariance of a single numerical value.

Table S24 summarizes empirical evidence from previous studies that report temporal stability, or limited temporal variation, in cross-sectional scaling exponents across different urban indicators.

**Table S24.** Empirical evidence for the temporal stability of cross-sectional scaling exponents.

| **Paper References** | **Response Indicators** | **Response Indicators** |
| --- | --- | --- |
| (Bettencourt, 2013) | Total wages | Analysis of total wages in US Metropolitan Statistical Areas (MSAs) over a 40-year period (1969–2008) found that exponents stayed consistent within their 95% confidence intervals, even as baseline wages grew considerably. |
| (Burghardt et al., 2024) | Developed area and road length | A 115-year study (1900–2015) of conterminous US cities found that scaling exponents for developed area and road length closely matched theoretical models (2/3 and 5/6, respectively) throughout the entire century. |
| (Chuqiao Yang, 2024) | Crime indicators | Scaling exponents for multiple categories of crime, including murder, rape, robbery, assault, and burglary, were reported to exhibit broadly consistent temporal patterns. |
| (Fragkias et al., 2013) | CO_2_ emissions | CO₂ emissions in US urban areas showed “remarkable stability” in scaling relationships between 1999 and 2008. |
| (Carleton et al., 2025) | Elite wealth | Comparative analysis of elite wealth indicators, including monuments and inscriptions in ancient Rome and billionaires and tall buildings in modern cities, found strikingly similar scaling parameters, typically between 0.3 and 0.5, despite a temporal gap of roughly 1,000 years. |
| (Depersin & Barthelemy, 2018) | Traffic delays | The scaling exponent of traffic delays fluctuated by approximately 20% between 1982 and 2014, but the relationship remained consistently superlinear over time. |
| (Xu et al., 2020) | Building areas | In Shanghai districts, cross-sectional scaling exponents for building areas varied modestly, from approximately 0.83 to 0.94, between 2005 and 2017. |

SI References

Arcaute, E., & Hatna, E. (2020). Scaling Laws: Insights and Limitations. In D. Pumain (Ed.), *Theories and Models of Urbanization: Geography, Economics and Computing Sciences* (pp. 45–66). Springer International Publishing. https://doi.org/10.1007/978-3-030-36656-8_4

Bettencourt, L. M. A. (2013). The Origins of Scaling in Cities. *Science*, *340*(6139), 1438–1441. https://doi.org/10.1126/science.1235823

Burghardt, K., Uhl, J. H., Lerman, K., & Leyk, S. (2024). Analyzing urban scaling laws in the United States over 115 years. *Environment and Planning B: Urban Analytics and City Science*, *51*(9), 2249–2263. https://doi.org/10.1177/23998083241240099

Carbone, A., Murialdo, P., Pieroni, A., & Toxqui-Quitl, C. (2022). Atlas of urban scaling laws. *Journal of Physics: Complexity*, *3*(2), 025007. https://doi.org/10.1088/2632-072X/ac718e

Carleton, W. C., Elton, H., Miranda, W., Work, I., Safarik, D., Winkelmann, R., Laubichler, M., Renn, J., & Roberts, P. (2025). Parallel scaling of elite wealth in ancient Roman and modern cities with implications for understanding urban inequality. *Nature Cities*, *2*(4), 344–355. https://doi.org/10.1038/s44284-025-00213-1

*Census Regions and Divisions of the United States*. (n.d.). Retrieved chrome-extension://efaidnbmnnnibpcajpcglclefindmkaj/https://www2.census.gov/geo/pdfs/maps-data/maps/reference/us_regdiv.pdf

CEU. JRC. (2019). *GHSL-OECD functional urban areas: Public release of GHS FUA.* Publications Office. https://data.europa.eu/doi/10.2760/67415

Chuqiao Yang, V. (2024). Mathematical model explaining variations in urban scaling exponents. In L. S. D’Acci, *Urban Scaling* (1st ed., pp. 187–194). Routledge. https://doi.org/10.4324/9781003288312-22

Depersin, J., & Barthelemy, M. (2018). From global scaling to the dynamics of individual cities. *Proceedings of the National Academy of Sciences*, *115*(10), 2317–2322. https://doi.org/10.1073/pnas.1718690115

Deru, M., Field, K., Studer, D., Benne, K., Griffith, B., Torcellini, P., Liu, B., Halverson, M., Winiarski, D., Rosenberg, M., Yazdanian, M., Huang, J., & Crawley, D. (2011). *U.S. Department of Energy Commercial Reference Building Models of the National Building Stock* (NREL/TP-5500-46861, 1009264; p. NREL/TP-5500-46861, 1009264). https://doi.org/10.2172/1009264

Florczyk, A., Corbane, C., Schiavina, M., Pesaresi, M., Maffenini, L., Melchiorri, M., Politis, P., Sabo, F., Freire, S., Ehrlich, D., Kemper, T., Tommasi, P., Airaghi, D., & Zanchetta, L. (2019). *GHS Urban Centre Database 2015, multitemporal and multidimensional attributes, R2019A* [Dataset]. European Commission, Joint Research Centre (JRC). https://doi.org/10.2905/53473144-b88c-44bc-b4a3-4583ed1f547e

Fragkias, M., Lobo, J., Strumsky, D., & Seto, K. C. (2013). Does Size Matter? Scaling of CO2 Emissions and U.S. Urban Areas. *PLOS ONE*, *8*(6), e64727. https://doi.org/10.1371/journal.pone.0064727

Frantz, D., Schug, F., Wiedenhofer, D., Baumgart, A., Virág, D., Cooper, S., Gomez-Medina, C., Lehmann, F., Udelhoven, T., Linden, S. van der, Hostert, P., & Haberl, H. (2023). *Material stock map of CONUS* (Version v. 1.1) [Dataset]. Zenodo. https://doi.org/10.5281/zenodo.8163466

Gately, C., Hutyra, L. R., & Wing, I. S. (2019). DARTE Annual On-road CO2 Emissions on a 1-km Grid, Conterminous USA, V2, 1980-2017. *ORNL DAAC*. https://doi.org/10.3334/ORNLDAAC/1735

Glaeser, E. L., & Kahn, M. E. (2010). The greenness of cities: Carbon dioxide emissions and urban development. *Journal of Urban Economics*, *67*(3), 404–418. https://doi.org/10.1016/j.jue.2009.11.006

Gurney, K. R., Liang, J., Patarasuk, R., Song, Y., Huang, J., & Roest, G. (2020). Vulcan: High-Resolution Annual Fossil Fuel CO2 Emissions in USA, 2010-2015, Version 3. *ORNL DAAC*. https://doi.org/10.3334/ORNLDAAC/1741

Hong, S., Hui, E. C. M., & Lin, Y. (2022). Relationships between carbon emissions and urban population size and density, based on geo-urban scaling analysis: A multi‑carbon source empirical study. *Urban Climate*, *46*, 101337. https://doi.org/10.1016/j.uclim.2022.101337

Jarmin, R. S. (n.d.). *Disclosure Avoidance for the 2020 Census: An Introduction*.

Louf, R., & Barthelemy, M. (2014). How congestion shapes cities: From mobility patterns to scaling. *Scientific Reports*, *4*(1), 5561. https://doi.org/10.1038/srep05561

Mendon, V., & Taylor, Z. T. (2014). *Development of residential prototype building models and analysis system for large-scale energy efficiency studies using Energyplus*. Building Simulation Conference.

Mohajeri, N., Gudmundsson, A., & French, J. R. (2015). CO2 emissions in relation to street-network configuration and city size. *Transportation Research Part D: Transport and Environment*, *35*, 116–129. https://doi.org/10.1016/j.trd.2014.11.025

Molinero, C., & Thurner, S. (2021). How the geometry of cities determines urban scaling laws. *Journal of The Royal Society Interface*, *18*(176), 20200705. https://doi.org/10.1098/rsif.2020.0705

Oliveira, E. A., Andrade, J. S., & Makse, H. A. (2014). Large cities are less green. *Scientific Reports*, *4*(1), Article 1. https://doi.org/10.1038/srep04235

PNNL. (2023). *Prototype Building Models*. https://www.energycodes.gov/prototype-building-models

Rao, Y., Zhong, Y., & He, Q. (2023). Evaluation of carbon emission efficiency based on urban scaling law: Take 308 cities in China as an example. *Environmental Science and Pollution Research*, *30*(48), 105166–105180. https://doi.org/10.1007/s11356-023-29634-y

Rybski, D., Reusser, D. E., Winz, A.-L., Fichtner, C., Sterzel, T., & Kropp, J. P. (2016). Cities as nuclei of sustainability? *Environment and Planning B: Urban Analytics and City Science*. (Sage UK: London, England). https://doi.org/10.1177/0265813516638340

Schiavina, M., Melchiorri, M., Pesaresi, M., Politis, P., Freire, S., Maffenini, L., Florio, P., Ehrlich, D., Goch, K., Tommasi, P., & Kemper, T. (2022). *GHSL data package 2022: Public release GHS P2022*. Publications Office of the European Union.

Schiavina, M., Moreno-Monroy, A., Maffenini, L., & Veneri, P. (2019). *GHS-FUA R2019A - GHS functional urban areas, derived from GHS-UCDB R2019A (2015)* [Dataset]. European Commission, Joint Research Centre (JRC). https://doi.org/10.2905/347F0337-F2DA-4592-87B3-E25975EC2C95

Schiavina, Marcello, Freire, Sergio, Alessandra Carioli, & MacManus, Kytt. (2023). *GHS-POP R2023A - GHS population grid multitemporal (1975-2030)* [Dataset]. https://doi.org/10.2905/2FF68A52-5B5B-4A22-8F40-C41DA8332CFE

Swanwick, R. H., Read, Q. D., Guinn, S. M., Williamson, M. A., Hondula, K. L., & Elmore, A. J. (2022). Dasymetric population mapping based on US census data and 30-m gridded estimates of impervious surface. *Scientific Data*, *9*(1), 523. https://doi.org/10.1038/s41597-022-01603-z

Taubenböck, H., Weigand, M., Esch, T., Staab, J., Wurm, M., Mast, J., & Dech, S. (2019). A new ranking of the world’s largest cities—Do administrative units obscure morphological realities? *Remote Sensing of Environment*, *232*, 111353. https://doi.org/10.1016/j.rse.2019.111353

the Population Reference Bureau & the U.S. Census Bureau’s 2020 Census Data Products and Dissemination Team. (n.d.). *Why the Census Bureau Chose Differential Privacy*.

the United States Census Bureau & the U.S. Department of Transportation. (2024). *Urban Areas* [esriGeometryPolygon]. https://geodata.bts.gov/datasets/usdot::urban-areas/about

United States Census Bureau. (n.d.). *Index of /geo/tiger/TIGER2020/TABBLOCK20*. Retrieved July 22, 2024, from https://www2.census.gov/geo/tiger/TIGER2020/TABBLOCK20/

Xu, G., Xu, Z., Gu, Y., Lei, W., Pan, Y., Liu, J., & Jiao, L. (2020). Scaling laws in intra-urban systems and over time at the district level in Shanghai, China. *Physica A: Statistical Mechanics and Its Applications*, *560*, 125162. https://doi.org/10.1016/j.physa.2020.125162
